# Diagnostic stewardship cutoffs for urinalysis results prior to performing a urine culture: analysis of data from a healthcare system

**DOI:** 10.1017/ice.2025.10265

**Published:** 2025-11

**Authors:** Deborah Kahler Kupferwasser, Amy Y. Kang, Michael Bolaris, Holly Huse, Liz Chen, Loren Miller

**Affiliations:** 1 Division of Infectious Diseases, The Lundquist Institute at Harbor-UCLA Medical Center, Torrance, CA, 90509, USA; 2 Department of Pharmacy Practice, Chapman University School of Pharmacy, Irvine, CA, USA; 3 Department of Pharmacy, Harbor-UCLA Medical Center, Torrance, CA, USA; 4 Los Angeles County, Department of Health Services, Rancho Los Amigos, Los Angeles CA, USA; 5 Los Angeles County, Department of Health Services, Harbor-UCLA Microbiology Laboratory, Los Angeles, CA, USA; 6 David Geffen School of Medicine at UCLA, Los Angeles, CA, USA

## Abstract

**Background:**

Urinary tract infections are commonly overdiagnosed. To minimize overdiagnosis, some laboratories utilize reflex algorithms that use urinalyses as preliminary screening before potentially proceeding to urine culture. However, the optimal urinalysis cutoffs for this diagnostic stewardship intervention remain poorly defined.

**Methods:**

We performed a retrospective, cross-sectional analysis from 2/1/21–1/31/23 in the Los Angeles County Department of Health Services healthcare system. We examined patient encounters in which urinalysis was ordered synchronously with urine culture. We categorized urine culture isolates as uropathogens or non-uropathogens. We calculated receiver operating characteristic curves of urinalysis parameters’ ability, singularly or in combination, to identify uropathogens.

**Results:**

Among 80,949 paired urinalysis and urine cultures (17,488 inpatient, 20,716 emergency department, 42,745 outpatient), cultures yielded 35% (*n* = 28,993) uropathogens, 4% (*n* = 2960) non-uropathogens, 37% (*n* = 29,951) contaminants, and 24% (*n* = 19,045) no growth. Among urinalysis parameters, white blood cells (WBCs) had the highest diagnostic accuracy (area under the curve (AUC)=0.722, [95% CI 0.718–0.725]), followed by leukocyte esterase (AUC = 0.700, [95% CI 0.690–0.701]), bacteria (AUC = 0.673, [95% CI 0.670–0.677]), nitrite (AUC = 0.627, [95% CI 0.625–0.630]), and squamous epithelial cells (AUC = 0.530, [95% CI 0.526–0.534]). WBC AUC values were consistent across healthcare settings (outpatient, emergency department, and inpatient). The urinalysis parameter combination with the highest AUC, WBC plus bacteria, performed worse than WBCs alone (AUC = 0.711 vs. AUC = 0.722, *p* = 0.001).

**Conclusion:**

WBC on microscopic urinalysis demonstrated the highest diagnostic accuracy for predicting uropathogens in urine cultures. Stewardship programs should consider prioritizing urinalysis WBC count as the screening tool to optimize urine culture utilization.

## Introduction

By 2050, an estimated 8.2 million deaths globally associated with antimicrobial-resistant infections will occur, an increase from 1.27 million deaths in 2019.^1,2^ By 2050, the added healthcare costs attributed to antimicrobial-resistant infections could reach an estimated 1 trillion US dollars.^3^ The emergence of antimicrobial-resistant pathogens is fueled by antibiotic use and misuse.^4^ Misdiagnosis and overdiagnosis of urinary tract infections (UTIs) is recognized as an important contributor to inappropriate antibiotic use.^5^

Misinterpretation of urine tests is a major driver of inappropriate UTI diagnosis.^6^ A positive urine culture without UTI-specific symptoms indicates asymptomatic bacteriuria, not a UTI, yet asymptomatic bacteriuria often leads to unnecessary antibiotic treatment.^5,7^ Among patients with asymptomatic bacteriuria, 32%–60% are inappropriately treated with antibiotics.^8,9^

Antibiotic stewardship programs are an essential part of hospital practice, aimed at optimizing antibiotic use.^10^ Diagnostic stewardship harnesses aspects of diagnostic testing to achieve stewardship goals, such as improving diagnostic accuracy and optimizing lab result output to minimize provider misinterpretation.^11^

A diagnostic stewardship approach to decrease inappropriate antibiotic prescribing for asymptomatic bacteriuria is the reflex urine culture. This algorithmic approach triggers a urine culture only if predefined urinalysis criteria are met, such as significant pyuria.^12^ Reflex urine cultures have been shown to reduce urine culture rates, subsequently reducing unnecessary antibiotic use.^13–17^ However, considerable variability exists in the urinalysis parameters and the thresholds used in reflex algorithms. A survey of 28 healthcare facilities that implemented a reflex urine culture program revealed 26 different reflex algorithms, highlighting the heterogeneity in approach.^18^

To address this heterogeneity, we aimed to evaluate the diagnostic performance of urinalysis parameters in predicting uropathogens from urine cultures across different patient care settings. We sought to identify the parameter(s) with the highest performance for detecting uropathogens, considering a wide range of urinalysis parameters and their combinations.

## Methods

### Study design

We conducted a retrospective, cross-sectional study of all synchronous urinalysis and urine culture tests performed within 24 hours of each other and ordered between 2/1/2021 and 1/31/2023 at the Los Angeles County Department of Health Services. This municipal safety net healthcare system consists of 26 ambulatory care centers and 4 acute care hospitals, primarily serving vulnerable populations. We examined all categories of patient encounters including inpatient, outpatient, and emergency department visits. We excluded encounters if the patients, based on demographics or associated ICD-10 codes, were under 3 months of age, had neutropenia, were pregnant, or were undergoing a urologic procedure, as screening or treatment of asymptomatic bacteriuria in these populations may be warranted.^5^

### Data collection

Patient data were retrieved from electronic medical records. We examined all episodes in which there were synchronous testing of urinalysis and urine culture. Synchronous was defined as tests ordered within the same calendar day, as previously described.^13,19^ Urinalysis and urine cultures were ordered by providers based on clinical judgment, and there was no system-wide protocol that automatically triggered urine culture performance based on urinalysis results. The specific clinical indication for testing, such as suspected UTIs, was unavailable in structured fields of the electronic medical record. At our institution, microscopic urinalysis is triggered based on predefined thresholds from the macroscopic urinalysis (eg, positive leukocyte esterase or nitrites), serving as an internal reflex mechanism. For this study, we limited our analysis to only those urinalysis and urine culture pairs in which microscopic analysis was performed. For each encounter, we collected demographic and healthcare encounter data, including age, gender, race, ethnicity, and healthcare setting (ie, outpatient, emergency department, or inpatient). From urinalysis macroscopic, we analyzed leukocyte esterase and nitrite tests performed using either Arkray AUTION MAX AX-4030 (Minneapolis, MN) or CLINITEK ATLAS® (Malvern, PA). For the microscopic examination, we analyzed white blood cells (WBCs), bacteria, and squamous epithelial cells using the Iris iQ200 Elite/Sprint (Santa Clara, CA) or Sysmex UF-1000i (Bohemia, NY). For urine cultures, results are classified into one of the following categories as defined by the clinical microbiology laboratory’s standard operating procedures: positive for growth, no growth, normal flora, or contaminated based on the growth of multiple (≥3) organisms.

### Data analysis

Our outcome of interest was uropathogen identification from urine culture results. Microorganisms isolated from urine cultures were categorized by two Infectious Disease specialists (LGM and AYK) into four categories: organisms usually considered uropathogens (eg, *Escherichia coli*), organisms sometimes considered uropathogens (eg, *Streptococcus anginosus*), organisms rarely considered uropathogens (eg, *Candida)*, or non-uropathogens of clinical significance (eg, *Salmonella* species, *S. aureus*, given its association with *S. aureus* bacteremia).^20^ For our analysis, a uropathogen was defined as any organism categorized as usually or sometimes considered a uropathogen or a non-uropathogen of clinical significance, as these results would likely prompt clinical action in the presence of UTI or infectious symptoms. Non-uropathogens were defined as microorganisms rarely considered uropathogens. Cultures were classified as “non-actionable” if they showed no growth, were contaminated, or grew non-uropathogens. For cultures with two organisms, one classified as a uropathogen and the other as a non-uropathogen, the culture was categorized as positive for a uropathogen.

We evaluated the diagnostic performance measures, sensitivity, specificity, and area under the curve (AUC) of the urinalysis parameters outlined above for predicting the presence of uropathogens. We conducted subgroup analysis across three major clinical settings: outpatient, emergency department, and inpatient, to assess potential differences in diagnostic performance among different patient populations. This stratification was based on our suspicion that patients’ ability to provide a clean catch urine specimen varies with illness severity, being lowest in the outpatient setting and highest in the inpatient setting.^21^ Additionally, we stratified analysis by age to compare pediatric and adult populations. AUC comparisons were performed using the DeLong statistic.

We generated receiver operating characteristic (ROC) curves for both individual urinalysis parameters and combined parameters to evaluate diagnostic performance. Combinations of urinalysis parameters were selected based on the method by which the tests are performed, either via macroscopic analysis or microscopically. This approach to combining urinalysis parameters allowed us to assess whether combining two higher-performing parameters could further improve diagnostic accuracy. All analyses were conducted using SAS software 9.4.^22^

## Results

During the study period, 80,949 urinalysis/urine culture pairs were identified. Mean age of our study population was 48 years (SD ± 19), and 69% were female. The most common patient race/ethnicity was Hispanic/Latino (69%), followed by White (12%) and Black/African American (10%) (Table 1). The frequency of urinalysis/urine cultures stratified by healthcare settings was: 53% (*n* = 42,745) in outpatient settings, 26% (*n* = 20,716) in the emergency department, and 22% (*n* = 17,488) inpatients. Urine sample collection method and culture results are presented in Supplemental Tables 1–3.

**Table 1.** Study population sample size and demographic descriptive statistics

Demographic characteristic	N (%) (total *n* = 80,949)
Gender	
Female	56,090 (69%)
Male	24,760 (31%)
Race/ethnicity	
Hispanic or Latino	55,942 (69%)
White	9650 (12%)
Black or African American	7726 (10%)
Asian or Asian American	3309 (4%)
Native Hawaiian or other Pacific Islander and American Indian or Alaska Native	3122 (4%)
Mixed race/other	647 (1%)
Decline to state	553 (1%)
Age (mean) years, ±standard deviation	48 ± 19

### Urinalysis results

Urinalysis results are summarized in Table 2. For nitrite, 87% (*n* = 70,387) of results were negative. The leukocyte esterase results were negative in 36% (*n* = 29,008) of cases. Results from microscopic urinalysis showed that the most common WBC count was 0–3 WBCs/High Power Field (HPF) in 36% (*n* = 28,867) of cases. Bacteria were most commonly classified as either few/HPF 45% (*n* = 36,087) or none/HPF 34% (*n* = 27,483). For squamous epithelial cells, the most frequent result was >30 squamous epithelial cells/Low Power Field (LPF) (36%, *n* = 29,368) (Table 2).

**Table 2.** Macroscopic and microscopic urinalysis parameter results from the study population

Urinalysis parameter	N (%) (total *n* = 80,949)
Macroscopic examination	
Nitrite	
Positive	10,562 (13%)
Negative	70,387 (87%)
Leukocyte esterase	
Negative	29,008 (36%)
Trace	8675 (11%)
Small	11,042 (14%)
Moderate	11,488 (14%)
Large	20,736 (26%)
Microscopic test	
WBC/HPF	
None	1509 (2%)
0–3	28,867 (36%)
4–10	17,428 (22%)
11–30	12,000 (15%)
31–50	4650 (6%)
>50	16,495 (20%)
Squamous epithelial cells/LPF	
None	14,702 (18%)
0–3	8763 (11%)
4–15	17,223 (21%)
16–30	10,893 (14%)
>30	29,368 (36%)
Bacteria/HPF	
None	27,483 (34%)
Few	36,087 (45%)
Moderate	8802 (11%)
Many	8577 (11%)

The percentage of tests excluded due to not having microscopic analysis was (*n* = 11,741,13%).

WBC, white blood cell; HPF, high power field; LPF, low power field.

**Figure 1.**
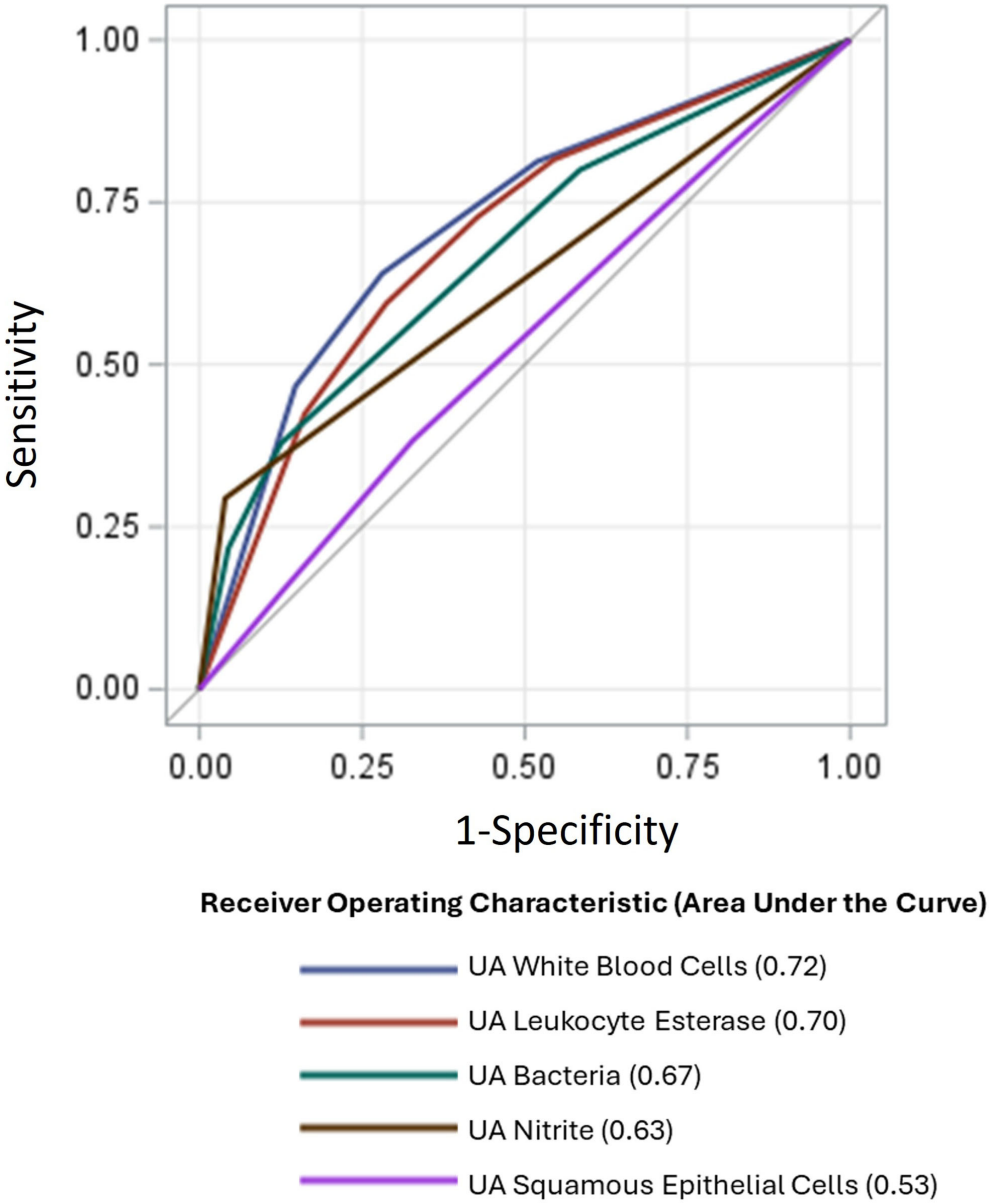
Receiver operating characteristic area under the curve for urinalysis parameters. UA, urinalayis.

Among microscopic urinalysis parameters, WBCs had the highest AUC for predicting uropathogens (0.722 [95% CI 0.718–0.725]), followed by bacteria (0.673 [95% CI 0.670–0.677]) and squamous epithelial cells (0.530 [95% CI 0.526–0.534]). For macroscopic urinalysis parameters, the AUC value for uropathogen prediction was 0.700 [95% CI 0.690–0.701], for leukocyte esterase, and 0.627 [95% CI 0.625–0.630]), for nitrite (Figure 1).

### Urinalysis parameter combinations

When WBCs, which had the highest AUC (0.722 [95% CI 0.718–0.725]) among urinalysis parameters, were combined with bacterial counts of “few” or more, the AUC was slightly lower (0.711[95% CI 0.706–0.714)). Combinations of WBCs with other microscopic parameters resulted in even lower AUCs (Table 3). Among the macroscopic parameter combinations, leukocyte esterase combined with nitrite (whether positive or negative) had an AUC of 0.695, which was identical to that of leukocyte esterase alone (AUC = 0.700 [95% CI 0.690–0.701], *p* = 0.08) but higher than nitrites alone (AUC = 0.627 [95% CI 0.625–0.630]), *p* = 0.001) (Table 3).

**Table 3.** Area under the curve values for individual and combined parameter microscopic and macroscopic urinalysis cutoffs stratified by hospital setting

Urinalysis cutoffs labs AUC [95% confidence intervals]	All clinical settings	Outpatient only (*n* = 42,754)	Emergency department only (*n* = 20,716)	Inpatient only (*n* = 17,488)
WBC	0.722 [0.718–0.725]	0.720 [0.715–0.725]	0.727 [0.719–0.733]	0.722 [0.714–0.730]
Leukocyte esterase	0.700 [0.690–0.701]	0.681 [0.676–0.686]	0.702 [0.694–0.708]	0.718 [0.711–0.726]
Bacteria	0.673 [0.670–0.677]	0.666 [0.661–0.671]	0.675 [0.668–0.682]	0.693 [0.686–0.702]
Nitrite	0.627 [0.625–0.630]	0.632 [0.628–0.635]	0.626 [0.621–0.632]	0.618 [0.612–0.624]
Squamous epithelial cells	0.530 [0.526–0.534]	0.539 [0.533–0.544]	0.520 [0.513–0.529]	0.531 [0.522–0.539]
WBC and bacteria				
WBC and ≥bacteria none seen	0.722 [0.718–0.725]	0.720 [0.715–0.725]	0.727 [0.719–0.733]	0.722 [0.714–0.729]
WBC and ≥bacteria few	0.711 [0.706–0.714]	0.709 [0.703–0.714]	0.716 [0.709–0.723]	0.714 [0.706–0.722]
WBC and ≥bacteria moderate	0.634 [0.631–0.637]	0.622 [0.618–0.626]	0.637 [0.631–0.643]	0.663 [0.656–0.670]
WBC and ≥bacteria many	0.587 [0.585–0.590]	0.575 [0.572–0.578]	0.591 [0.586–0.596]	0.614 [0.608–0.620]
WBC and squamous epithelial cells				
WBC & Squamous epithelial cells 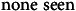	0.722 [0.717–0.725]	0.720 [0.715–0.730]	0.727 [0.719–0.733]	0.722 [0.714–0.730]
WBC and squamous epithelial cells 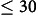	0.689 [0.685–0.692]	0.680 [0.675–0.685]	0.695 [0.688–0.702]	0.711 [0.704–0.720]
WBC and squamous epithelial cells 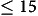	0.665 [0.662–0.670]	0.646 [0.641–0.651]	0.677 [0.671–0.685]	0.700 [0.693–0.709]
WBC and squamous epithelial cells 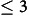	0.611 [0.609–0.616]	0.588 [0.585–0.593]	0.623 [0.617–0.630]	0.650 [0.645–0.660]
WBC and squamous epithelial cells 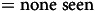	0.579 [0.572–0.583]	0.563 [0.560–0.567]	0.587 [0.583–0.594]	0.607 [0.603–0.616]
Urinalysis cutoffs macroscopic labs				
Leukocyte esterase and nitrite				
Leukocyte esterase ≥ − and nitrite − or +	0.695 [0.691–0.700]	0.681 [0.676–0.690]	0.701 [0.694–0.708]	0.719 [0.711–0.727]
Leukocyte esterase ≥ − and nitrite +	0.628 [0.625–0.631]	0.632 [0.628–0.636]	0.627 [0.622–0.633]	0.620 [0.613–0.630]

AUC, area under the curve; WBC, white blood cell.

### Results by healthcare settings

The AUC for urinalysis WBC as a predictor for uropathogen prediction was consistent across settings, (*P* > 0.05 for all comparisons, Supplemental Table 4): 0.720 [95% CI 0.715–0.725] in the outpatient setting, 0.727 [95% CI 0.714–0.730] in the emergency department, and 0.722 [95% CI 0.714–0.730] in the inpatient setting (Table 3). Additional results of other urinalysis parameters are summarized in Table 3.

The diagnostic accuracy of combined macroscopic urinalysis parameters stratified by healthcare setting is presented in Table 3. The AUC of combining a positive leukocyte esterase result with nitrite test of any result as a predictor of detecting a uropathogen was 0.681 [95% CI 0.676–0.690] in the outpatient setting, 0.701 [95% CI 0.694–0.708] in the emergency department, and 0.719 [95% CI 0.711–0.727] in the inpatient setting. These AUC values were lower than the values obtained from combining WBC results with any bacteria result, which were 0.720 [95% CI 0.715–0.725], 0.727 [95% CI 0.719–0.733], and 0.722 [95% CI 0.714–0.729] for the outpatient setting (*p* < 0.001), emergency department (*p* < 0.001), and inpatient setting (*p* = 0.60), respectively.

### Results by age group

Stratifying our pediatric population into different age categories showed that WBCs as a predictor of detecting a uropathogen had the highest AUC expect the very young (3 months–2 years), in which leukocyte esterase performed best (AUC = 0.739 [95% CI 0.693–0.786], Supplemental Table 5). Full age stratified results are similar to the overall population and outlined in Supplemental Tables 5 and 6.

### WBC cutoffs and urine cultures performed and prevented

**Figure 2.**
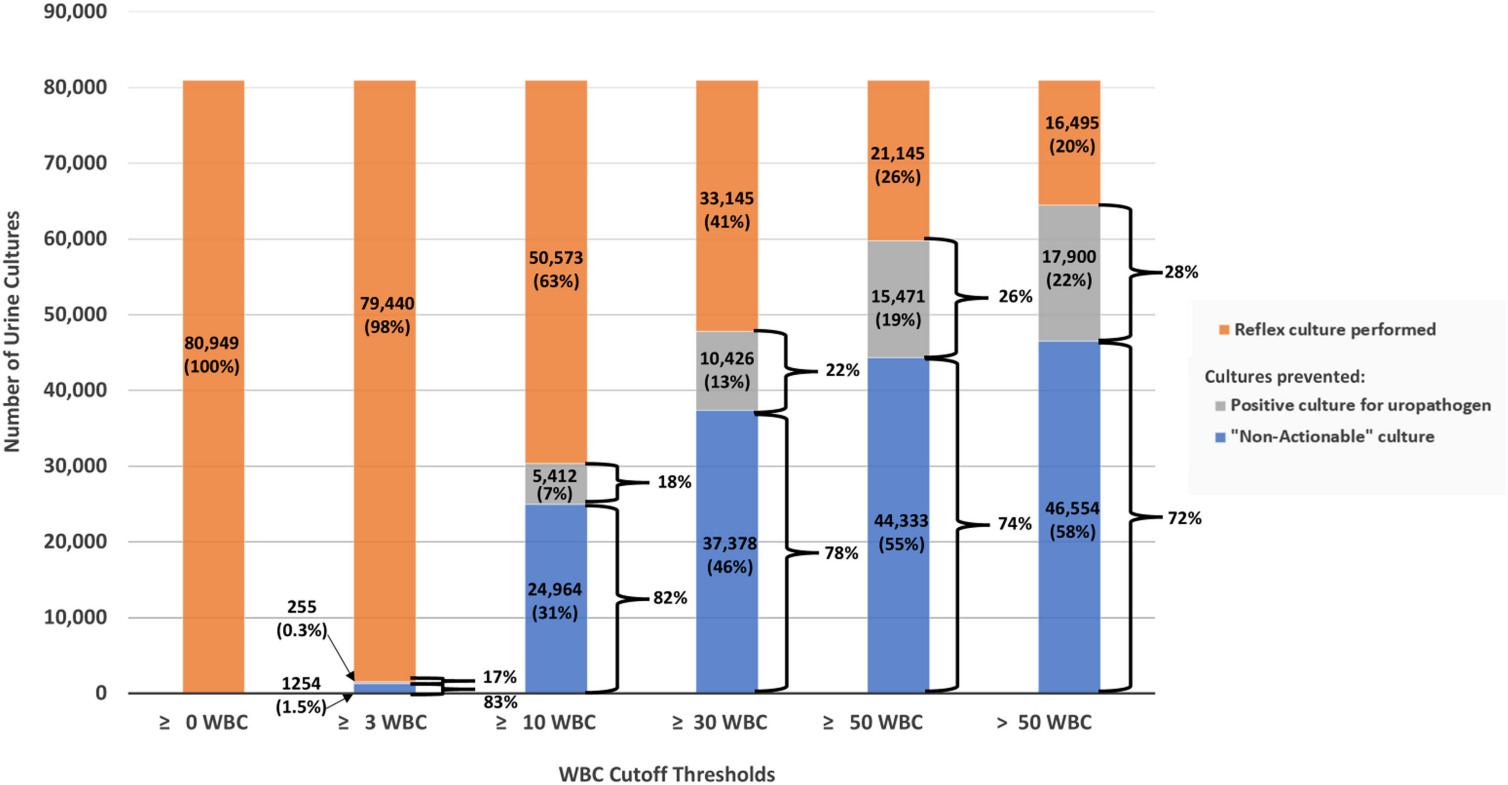
Number of urine cultures performed and prevented at each white blood cell cutoff threshold. WBC, white blood cell.

We graphically represented the expected results of applying various WBC cutoffs, the urinalysis parameter with the highest AUC value (Figure 2). Without a cutoff, 100% (*n* = 80,949) cultures would be performed. In contrast, using a cutoff of >50 WBCs/HPF, only 20% of urine cultures would be performed (*n* = 16,495). Additionally, with increasing WBC cutoff thresholds, the number of “non-actionable cultures” prevented also increased, from 1.5% (*n* = 1254) of total cultures at a cutoff of ≥ 3 WBCs/HPF to 58% of total cultures (*n* = 46,554) at a cutoff of > 50 WBCs/HPF (Figure 2). Similarly, among all urine culture (*n* = 80,949), the number of cultures positive for a uropathogen that would have been missed with reflex testing rose from *n* = 255 (0.3% of all urine tests) at a cutoff of ≥3 WBC/HPF to *n* = 17,900 (22%) at a cutoff of >50 WBCs/HPF (Figure 2). Among the urine cultures prevented, as the proportion of “non-actionable cultures” decreased with WBC cutoffs, from 83% at a cutoff of ≥ 3 WBCs /HPF to 72% at the highest threshold. The percent of cultures positive for a uropathogen missed increased from 17% to 28% as the WBC cutoff rose (Figure 2). When examining the proportion of positive urine cultures for a uropathogen prevented (and excluding the ≥3 WBC cutoff, which only excluded 1.8% of urine cultures from being done), the ≥10 WBCs/HPF show the lowest proportion of “positive urine cultures for a uropathogen” prevented (18%) when compared to the higher WBCs cutoff thresholds (22–28%) (Figure 2).

### Sensitivity and specificity tables: urinalysis individual and combined results

Sensitivity and specificity of urinalysis results for different cutoffs of urinalysis parameters are shown in Table 4. As WBC values increase, sensitivity for detecting a uropathogen decreases, conversely, specificity increases (Table 4). For example, using a WBC cutoff of ≥ 10 WBCs/HPF, 62% (*n* = 50,573) urine cultures would be processed, with 19% (*n* = 5412) of uropathogen-positive cultures and 32% (*n* = 947) of non-uropathogen cultures excluded from processing (Table 4). The combination of urine microscopic WBCs and bacteria showed similar trends in sensitivity and specificity as WBCs alone (Supplemental Table 7). At a cutoff of ≥10 WBCs/HPF and ≥ bacteria few, 51% (*n* = 40,843) urine cultures would be processed. Using this cutoff, 29% (*n* = 8372) of cultures that grew uropathogens and 45% (*n* = 1333) of cultures that grew non-uropathogens would be excluded from processing (Supplemental Table 5). Additional urinalysis combination results for sensitivity and specificity analysis are shown in Supplemental Tables 8 and 9.

**Table 4.** Sensitivity, specificity, and negative predictive value for urine culture reflex cutoff values for urinalysis microscopic and macroscopic tests (*n* = 80,949). Additional columns include results for processed and excluded urine cultures at each urine culture reflex cutoff values for both uropathogens and non-uropathogens

WBC count on urine microscopy (WBCs/HPF) AUC = 0.722	Sensitivity for detecting a uropathogen *n* = 28,993	Specificity for detecting a uropathogen *n* = 51,956	Negative predictive value for detecting a non-uropathogen that is a true non-uropathogen	% of ordered urine cultures that would be processed *n* = 80,949	% of uropathogens that would be excluded from culture *n* = 28,993	% of non-uropathogens that would be excluded from culture *n* = 2960
≥ 0 WBC	100% (28,993)	0% (0)		100% (80,949)	0% (0)	0% (0)
≥ 3 WBC	99% (28,738)	2% (1254)	83% (1254/1509)	98% (79,440)	1% (255)	1% (23)
≥ 10 WBC	81% (23,581)	48% (24,964)	82% (24,964/30,376)	62% (50,573)	19% (5412)	32% (947)
≥ 30 WBC	64% (18,567)	72% (37,378)	78% (37,378/47,804)	41% (33,145)	36% (10,426)	57% (1673)
≥ 50 WBC	47% (13,522)	85% (44,333)	74% (44,333/59,804)	26% (21,145)	53% (15,471)	73% (2160)
> 50 WBC	38% (11,93)	90% (46,554)	72% (46,554/64,454)	20% (16,495)	62% (17,900)	80% (2,363)

Bacteria count on urine microscopy (bacteria/HPF) AUC = 0.673	Sensitivity for detecting a uropathogen *n* = 28,993	Specificity for detecting a uropathogen *n* = 51,956	Negative predictive value for detecting a non-uropathogen that is a true non-uropathogen	% of ordered urine cultures that would be processed *n* = 80,949	% of uropathogens that would be excluded from culture *n* = 28,993	% of non-uropathogens that would be excluded from culture *n* = 2,960
≥ Bacteria none seen	100% (28,993)	0% (0)		100% (80,949)	0% (0)	0% (0)
≥ Bacteria few	80% (23,162)	42% (21,652)	79% (21,652/27,483)	66% (53,466)	20% (5,831)	28% (827)
≥ Bacteria moderate	38% (10,926)	88% (45,503)	72% (45,503/63,570)	22% (17,379)	62% (18,67)	80% (2364)
≥ Bacteria many	22% (6277)	96% (49,656)	69% (49,656/72,372)	11% (8577)	78% (22,716)	92% (2768)

Nitrite on urine macroscopic examination AUC = 0.627	Sensitivity for detecting a uropathogen *n* = 28,993	Specificity for detecting a uropathogen *n* = 51,956	Negative predictive value for detecting a non-uropathogen that is a true non-uropathogen	% of ordered urine cultures that would be processed *n* = 80,949	% of uropathogens that would be excluded from culture *n* = 28,993	% of non-uropathogens that would be excluded from culture *n* = 2,960
Nitrite - and +	100% (28,993)	0% (0)		100% (80,949)	0% (0)	0% (0)
Nitrite +	29% (8522)	96% (49,916)	71% (49,916/70,387)	13% (10,562)	71% (20,471)	94% (2775)

Leukocyte esterase on urine macroscopic examination AUC = 0.700	Sensitivity for detecting a uropathogen *n* = 28,993	Specificity for detecting a uropathogen *n* = 51,956	Negative predictive value for detecting a non-uropathogen that is a true non-uropathogen	% of ordered urine cultures that would be processed *n* = 80,949	% of uropathogens that would be excluded from culture *n* = 28,993	% of non-uropathogens that would be excluded from culture *n* = 2,960
≥ Leukocyte esterase -	100% (28,993)	0% (0)		100% (80,949)	0% (0)	0% (0)
≥ Leukocyte esterase trace	82% (23,645)	46% (23,660)	82% (23,660/29,008)	64% (51,941)	18% (5396)	29% (872)
≥ Leukocyte esterase small	73% (21,71)	57% (29,761)	79% (29,761/37,683)	54% (43,266)	27% (7922)	40% (1192)
≥ Leukocyte esterase moderate	59% (17,268)	71% (0,37)	76% (37,000/48,725)	40% (32,224)	41% (11,725)	56% (1645)
≥ Leukocyte esterase large	42% (12,326)	84% (43,546)	72% (43,546/60,213)	26% (20,736)	58% (16,667)	72% (2118)

WBC, white blood cell; ROC, receiver operating characteristics curve; AUC; area under the curve.

Examining sensitivity and specificity by care setting, for samples collected in the outpatient setting, applying a urinalysis cutoff of ≥ 10 WBCs/HPF and ≥ bacteria classified as few or more resulted in 49% (*n* = 20,822) of urine cultures processed (Supplemental Tables 10–18). Using this cutoff, 31% (*n* = 4711) of cultures that grew uropathogens and 49% (*n* = 707) cultures that grew non-uropathogens would be excluded from processing. Results from the emergency department and inpatient setting are summarized in Supplemental Tables 10–18.

## Discussion

The growing incidence of infections caused by antimicrobial-resistant bacteria poses a significant public health threat, driven heavily by inappropriate antibiotic use.^23^ Diagnostic stewardship, including tools like reflex urine culture, can help reduce unnecessary testing and subsequent unwarranted antibiotic treatments, though optimal thresholds are not well-defined. In our analysis of urinary parameters, urine microscopy WBC count demonstrated the highest diagnostic accuracy for predicting the presence of uropathogens.

Our study findings show that no single urinalysis parameter achieves both high sensitivity and specificity for detecting uropathogens from urine culture. However, WBC count on the microscopic portion of the urinalysis showed the best test performance with an AUC of 0.72, indicating acceptable or fair discriminative power.^24,25^

Our results align with an analysis of University of Pittsburgh Medical Center healthcare system data, which assessed urinalysis parameters for diagnostic accuracy of “significant bacteriuria”, a proxy definition for UTI. They found that WBC had the best diagnostic accuracy (AUC = 0.79), followed by leukocyte esterase (AUC = 0.78) and bacteria (AUC = 0.77).^15^ Interestingly, our AUC values were lower. Differences may stem from test ordering practice variation, with some systems performing urinalysis with urine cultures more or less discriminately, including ordering tests for patients without UTI symptoms.^26^ The bias introduced by selecting patients based on clinician-initiated urinalysis ordering, rather than on symptoms, has been shown to underestimate diagnostic accuracy and may have contributed to inter-study variability in AUC results.^27^ Another key difference is our mean population age was lower (48 versus 57 years), attributed to our inclusion of children, whereas the other study included only adults. Increased age is associated with a higher prevalence of pyuria and colonization in women,^28–30^ which might contribute to test performance differences. Finally, uropathogen definition differences may explain discrepant AUC values. The previous study’s outcome was >10,000 colony forming units (CFU)/mL, whereas our definition was organisms likely to prompt clinical action.

Unlike some previous investigations that combined macroscopic and microscopic urinalysis results and did not stratify results by hospital setting,^13,31^ we examined potential healthcare setting differences. Surprisingly, our results show combining urinalysis parameters did not improve diagnostic accuracy, in contrast to results from Ourani et al.^32^ The variance may be explained by their definition of a positive urine culture, which included all positive cultures, and thus likely included non-uropathogens.^32^ Our results, for example, show WBCs combined with bacteria did not demonstrate better performance (AUC 0.711 95% CI 0.706-0.714]), compared to WBCs alone (AUC = 0.722 [95% CI 0.718-0.725], *P* = 0.001), suggesting that single, high-performing parameters like WBCs may be sufficient for reflex urine culture workflows, rather than more complex algorithms that do not provide additional value. Furthermore, we did not find a care setting (outpatient, emergency department, and inpatient) with clinically meaningful differences in urinalysis parameter test performance. Therefore, our data suggests no care site-specific urine reflex algorithm is warranted.

Our study evaluated the diagnostic accuracy of squamous epithelial cells in predicting uropathogens. Although many experts consider squamous epithelial cells as a marker for contaminated urine cultures,^13,31,33^ our study is among the few to examine their potential use in a urine reflex algorithm. Our results suggest that squamous epithelial cells are poor predictors of uropathogens (AUC = 0.53), consistent with Maher et al., who similarly found squamous epithelial cells unhelpful for reflex urine algorithms.^33^

Our study has strengths. One is our large sample size urinalysis-urine culture pairs (*n* = 80,949). Although ours is not the largest study in this area, our sample size exceeds that of the University of Pittsburgh group (*n* = 19,511)^15^ and is second to only to Advani et al.’s 221,963 pairs.^13^ Unlike Advani et al., who grouped data from inpatient and outpatient settings, our study took a more granular approach by stratifying healthcare settings.

Our study has limitations. The urinalysis parameters were assessed to identify uropathogens rather than to diagnose UTIs clinically. UTI diagnosis requires the presence of symptoms (eg, dysuria), which were not analyzed in this study given patient symptoms were in nonformatted text within the patient’s medical record. Although it may be ideal to include clinical symptomatology in conjunction with urinalysis parameters to refine diagnostic accuracy further, our primary focus was on diagnostic stewardship, specifically examining how clinical microbiology laboratories could reduce unnecessary urine cultures while minimizing non-performance of urine specimens that may represent true infections, consistent with prior similar studies.^13,15,34–36^ Additionally, quantitative information about urine culture colony growth was unavailable for our analysis due to information not present in structured fields within the electronic medical record. Although the use of quantitative thresholds is typically used when defining positive urine cultures, it should be noted that colony counts down to 1000 CFU/mL can represent a UTI.^37^ Another limitation is that our analysis only included urinalysis specimens that had microscopic analysis. However, urinalysis without microscopy occurs largely when urinalysis leukocyte is negative, suggesting that our analysis excludes patients unlikely to have a UTI. Lastly, our patient population consists of a safety net population in Southern California, which may not reflect care delivery patterns or patient demographics in other locales.

In conclusion, our findings support the use of WBC as the most reliable parameter for reflex urine culture algorithms. This single, simple parameter performs reasonably well in common care settings and across different ages. Our analysis also provides guidance as to expectations of urinary cultures performed and prevented when various WBC cutoffs are implemented. Our findings suggest that a cutoff of 10 WBCs/HPF, as suggested by others,^38^ may be a “sweet spot” that prevents a sizable proportion of urine cultures that do not yield uropathogens with minimal impact for isolating uropathogens. Future studies examining patient outcomes and safety after reflex urine culture algorithm implementation in diverse patient populations and settings will provide important information about its value in real world settings.

## Supporting information

Kupferwasser et al. supplementary material Kupferwasser et al. supplementary material

## Supplementary material

To view supplementary material for this article, please visit https://doi.org/10.1017/ice.2025.10265
