# Supplementary material for: Diagnostic stewardship cutoffs for urinalysis results prior to performing a urine culture: analysis of data from a healthcare system
Source: Infect Control Hosp Epidemiol. 2025 Sep 16;46(11):1097–104. doi: 10.1017/ice.2025.10265 (PMC12620065; doi:10.1017/ice.2025.10265)
Supplement: Kupferwasser et al. supplementary material [file S0899823X25102651sup001.docx]

Supplemental Table 1: Classification of Pathogenicity of Microorganisms from Urine Cultures

| **Microorganism Classification*** | N (%)  (Total n =80,949) |
| --- | --- |
|  |  |
| Organisms usually considered uropathogens | 24,383 (30%) |
| Organisms sometimes considered uropathogens | 4,320 (5%) |
| Organisms rarely considered uropathogens | 2,960 (4%) |
| Non-uropathogens of clinical significance | 290 (0.5%) |
| No Growth | 19,045 (24%) |
| Contamination | 29,951 (37%) |

Supplemental Table 1 Legend

*For definitions of each category, please see text.

Positive growth is defined as an organism identified as belonging to one of four categories: 1. Organisms usually considered uropathogens, 2. Organisms sometimes considered uropathogens. 3. organisms rarely considered uropathogens, and 4. non-uropathogens of clinical significance. Normal flora are bacteria found in low numbers (less than 10,000 CFU/ml such as *E. coli*). Urine cultures were defined as contaminated if 3 or more organisms were identified

Supplemental Table 2. Frequency of Microorganism Identified from Urine Culture Results

| **Microorganism** | **% (N)** |
| --- | --- |
| Escherichia coli | 55% (15,807) |
| Klebsiella pneumoniae | 9.6 % (2,795) |
| Gram Negative Rods | 9.2% (2,658) |
| Enterococcus species | 5.8% (1,679) |
| Proteus species | 5.5 % (1,597) |
| Pseudomonas species | 2.5% (714) |
| Enterobacter species | 1.5% (442) |
| other | 11.4% (3,301) |

Supplemental Table 3. Urine Specimen Collection Method for the Study Population

| **Specimen collection method** | **N (%)**  **(Total n =80,949)** |
| --- | --- |
| Clean Catch | 70,596 (87%) |
| Foley | 6,638 (8%) |
| Ileal Conduit | 81 (0.1%) |
| Cystoscopy | 486 (0.6%) |
| Condom Cath | 275 (0.34%) |
| In/Out Cath | 2,428 (3%) |
| Nephrostomy | 267 (0.3%) |
| Pedibag | 81 (0.1%) |
| Suprapubic | 97 (0.1%) |

Supplemental Table 4. Macroscopic and Microscopic Urinalysis Parameters both Singular and Combined Area Under the Curve Comparisons Between Different Hospital Settings are Shown with Associated z-scores and p-values.

| Urinalysis parameter | Hospital Setting | z score | p-value |
| --- | --- | --- | --- |
| WBC | Inpatient vs Outpatient | 0.233 | 0.82 |
| WBC | ED vs Outpatient | 1.811 | 0.07 |
| WBC | ED vs Inpatient | 0.205 | 0.84 |
| Leukocyte esterase | Inpatient vs Outpatient | 7.400 | <0.0001 |
| Leukocyte esterase | ED vs Outpatient | 7.400 | <0.0001 |
| Leukocyte esterase | ED vs Inpatient | 6.541 | <0.0001 |
| Bacteria | Inpatient vs Outpatient | 4.77 | <0.0001 |
| Bacteria | ED vs Outpatient | 2.25 | 0.0244 |
| Bacteria | ED vs Inpatient | 4.773 | <0.0001 |
| Nitrite | Inpatient vs Outpatient | 3.883 | <0.0001 |
| Nitrite | ED vs Outpatient | 2.000 | 0.05 |
| Nitrite | ED vs Inpatient | 3.30 | 0.001 |
| Squamous epithelial cells | Inpatient vs Outpatient | 1.414 | 0.16 |
| Squamous epithelial cells | ED vs Outpatient | 4.500 | <0.0001 |
| Squamous epithelial cells | ED vs Inpatient | 1.414 | 0.16 |
| WBC & >Bacteria none seen | Inpatient vs Outpatient | 0.400 | 0.69 |
| WBC & >Bacteria none seen | ED vs Outpatient | 1.750 | 0.08 |
| WBC & >Bacteria none seen | ED vs Inpatient | 0.353 | 0.72 |
| WBC & >Bacteria few | Inpatient vs Outpatient | 1.00 | 0.32 |
| WBC & >Bacteria few | ED vs Outpatient | 1.750 | 0.08 |
| WBC & >Bacteria few | ED vs Inpatient | 0.884 | 0.38 |
| WBC & >Bacteria moderate | Inpatient vs Outpatient | 9.168 | <0.0001 |
| WBC & >Bacteria moderate | ED vs Outpatient | 5.00 | <0.0001 |
| WBC & >Bacteria moderate | ED vs Inpatient | 8.200 | <0.0001 |
| WBC & >Bacteria many | Inpatient vs Outpatient | 10.82 | <0.0001 |
| WBC & >Bacteria many | ED vs Outpatient | 2.667 | 0.01 |
| WBC & >Bacteria many | ED vs Inpatient | 5.814 | <0.0001 |
| WBC & Squamous epithelial cells ≥ none seen | Inpatient vs Outpatient | 0.400 | 0.69 |
| WBC & Squamous epithelial cells ≥ none seen | ED vs Outpatient | 1.750 | 0.08 |
| WBC & Squamous epithelial cells ≥ none seen | ED vs Inpatient | 0.354 | 0.72 |
| WBC & Squamous epithelial cells ≤ 30 | Inpatient vs Outpatient | 6.200 | <0.0001 |
| WBC & Squamous epithelial cells ≤ 30 | ED vs Outpatient | 3.75 | 0.0002 |
| WBC & Squamous epithelial cells ≤ 30 | ED vs Inpatient | 5.480 | <0.0001 |
| WBC & Squamous epithelial cells ≤ 15 | Inpatient vs Outpatient | 10.800 | <0.0001 |
| WBC & Squamous epithelial cells ≤ 15 | ED vs Outpatient | 7.750 | <0.0001 |
| WBC & Squamous epithelial cells ≤ 15 | ED vs Inpatient | 9.546 | <0.0001 |
| WBC & Squamous epithelial cells ≤ 3 | Inpatient vs Outpatient | 13.863 | <0.0001 |
| WBC & Squamous epithelial cells ≤ 3 | ED vs Outpatient | 11.667 | <0.0001 |
| WBC & Squamous epithelial cells ≤ 3 | ED vs Inpatient | 12.400 | <0.0001 |
| WBC & Squamous epithelial cells = none seen | Inpatient vs Outpatient | 12.203 | <0.0001 |
| WBC & Squamous epithelial cells = none seen | ED vs Outpatient | 8.00 | <0.0001 |
| WBC & Squamous epithelial cells = none seen | ED vs Inpatient | 10.370 | <0.0001 |
| Leukocyte esterase > - & Nitrite - or + | Inpatient vs Outpatient | 7.600 | <0.0001 |
| Leukocyte esterase > - & Nitrite - or + | ED vs Outpatient | 6.718 | <0.0001 |
| Leukocyte esterase > - & Nitrite - or + | ED vs Inpatient | 7.600 | <0.0001 |
| Leukocyte esterase > - & Nitrite + | Inpatient vs Outpatient | 3.328 | 0.001 |
| Leukocyte esterase > - & Nitrite + | ED vs Outpatient | 2.828 | 0.01 |
| Leukocyte esterase > - & Nitrite + | ED vs Inpatient | 3.328 | 0.001 |

Supplemental Table 4 Legend

Abbreviations:

ED= Emergency department

WBC=White blood cell

Supplemental Table 5. Individual and Combined Microscopic and Macroscopic Urinalysis Cutoffs Area Under the Curve (AUC) Values with Confidence Intervals, Stratified by **Age Group Catigories**.

| **Urinalysis Cutoffs Labs**  **AUC**  **[95% Confidence Intervals]** | **Age**  **3 months-**  **2 years**  **(n=484)** | **Age**  **3-5 years**  **(n=2005)** | **Age**  **6-18 years**  **(n=1567)** | **Age**  **>18 years**  **(n=76,893)** |
| --- | --- | --- | --- | --- |
| WBC | 0.734  [0.684-0.783] | 0.725  [0.702-0.748] | 0.710  [0.682-0.739] | 0.721  [0.717-0.725] |
| Leukocyte esterase | 0.739  [0.693-0.786] | 0.694  [0.670-0.717] | 0.670  [0.641-0.700] | 0.694  [0.691-0.698] |
| Bacteria | 0.687  [0.635-0.738] | 0.713  [0.691-0.736] | 0.643  [0.614-0.673] | 0.672  [0.669-0.676] |
| Nitrite | 0.576  [0.544-0.607] | 0.631  [0.614-0.648] | 0.634  [0.612-0.656] | 0.627  [0.624-0.630] |
| Squamous epithelial cells | 0.578  [0.523-0.632] | 0.529  [0.503-0.554] | 0.517  [0.488-0.547] | 0.530  [0.529-0.534] |
| **WBC & Bacteria** |  |  |  |  |
| WBC & >Bacteria none seen | 0.734  [0.684-0.783] | 0.725  [0.702-0.748] | 0.710  [0.682-0.739] | 0.721  [0.717-0.725] |
| WBC & >Bacteria few | 0.725  [0.673-0.779] | 0.734  [0.711-0.757] | 0.695  [0.665-0.724] | 0.710  [0.706-0.714] |
| WBC & >Bacteria moderate | 0.623  [0.584-0.662] | 0.647  [0.629-0.665] | 0.630  [0.605-0.654] | 0.633  [0.630-0.637] |
| WBC & >Bacteria many | 0.565  [0.536-0.595] | 0.591  [0.576-0.601] | 0.582  [0.563-0.601] | 0.587  [0.585-0.590] |
| **WBC & Squamous epithelial cells** |  |  |  |  |
| WBC & Squamous epithelial cells $\geq none seen$ | 0.734  [0.684-0.783] | 0.725  [0.702-0.748] | 0.710  [0.682-0.739] | 0.721  [0.717-0.725] |
| WBC & Squamous epithelial cells $\leq30$ | 0.726  [0.676-0.776] | 0.719  [0.696-0.743] | 0.678  [0.650-0.706] | 0.687  [0.683-0.691] |
| WBC & Squamous epithelial cells $\leq15$ | 0.727  [0.676-0.778] | 0.723  [0.699-0.476] | 0.647  [0.621-0.673] | 0.663  [0.660-0.667] |
| WBC & Squamous epithelial cells $\leq3$ | 0.724  [0.690-0.789] | 0.694  [0.671-0.717] | 0.596  [0.575-0.617] | 0.608  [0.605-0.611] |
| WBC & Squamous epithelial cells $=none seen$ | 0.705  [0.657-0.754] | 0.652  [0.631-0.674] | 0.572  [0.554-0.591] | 0.576  [0.573-0.578] |
| **Urinalysis Cutoffs Macroscopic Labs** |  |  |  |  |
| **Leukocyte esterase & Nitrite** |  |  |  |  |
| Leukocyte esterase > - & Nitrite - or + | 0.740  [0.693-0.786] | 0.694  [0.670-0.717] | 0.670  [0.641-0.700] | 0.694  [0.691-0.698] |
| Leukocyte esterase > - & Nitrite + | 0.576  [0.544-0.607] | 0.632  [0.615-0.648] | 0.635  [0.613-0.656] | 0.628  [0.625-0.631] |

Supplemental Table 6. Macroscopic and Microscopic Urinalysis Parameters Both Singular and Combined Area Under the Curve Comparisons Between Different Age Group Categories are Shown with Associated z-scores and p-values.

| Test | Age Group Category | z score | p-value |
| --- | --- | --- | --- |
| WBC | 3 months-2 years vs  3-5 years | 0.279 | 0.78 |
| WBC | 3 months-2 years vs  6-18 years | 0.716 | 0.47 |
| WBC | 3 months-2 years vs  >18 years | 0.499 | 0.62 |
| WBC | 3-5 years vs  6-18 years | 0.781 | 0.43 |
| WBC | 3-5 years vs  >18 years | 0.329 | 0.74 |
| WBC | 6-18 years vs  >18 years | 0.992 | 0.32 |
| Leukocyte esterase | 3 months-2 years vs  3-5 years | 1.393 | 0.16 |
| Leukocyte esterase | 3 months-2 years vs  6-18 years | 2.057 | 0.04 |
| Leukocyte esterase | 3 months-2 years vs  >18 years | 0.780 | 0.42 |
| Leukocyte esterase | 3-5 years vs  6-18 years | 1.249 | 0.21 |
| Leukocyte esterase | 3-5 years vs  >18 years | 0.329 | 0.74 |
| Leukocyte esterase | 6-18 years vs  >18 years | 0.991 | 0.32 |
| Bacteria | 3 months-2 years vs  3-5 years | 0.893 | 0.37 |
| Bacteria | 3 months-2 years vs  6-18 years | 1.493 | 0.14 |
| Bacteria | 3 months-2 years vs  >18 years | 2.684 | 0.01 |
| Bacteria | 3-5 years vs  6-18 years | 3.763 | 0.0002 |
| Bacteria | 3-5 years vs  >18 years | 3.667 | 0.0002 |
| Bacteria | 6-18 years vs  >18 years | 0.991 | 0.32 |
| Nitrite | 3 months-2 years vs  3-5 years | 3.075 | 0.002 |
| Nitrite | 3 months-2 years vs  6-18 years | 3.074 | 0.002 |
| Nitrite | 3 months-2 years vs  >18 years | 0.186 | 0.85 |
| Nitrite | 3-5 years vs  6-18 years | 0.234 | 0.81 |
| Nitrite | 3-5 years vs  >18 years | 12.283 | <0.0001 |
| Nitrite | 6-18 years vs  >18 years | 0.982 | 0.33 |
| Squamous epithelial cells | 3 months-2 years vs  3-5 years | 1.826 | 0.07 |
| Squamous epithelial cells | 3 months-2 years vs  6-18 years | 2.155 | 0.03 |
| Squamous epithelial cells | 3 months-2 years vs  >18 years | 0.498 | 0.69 |
| Squamous epithelial cells | 3-5 years vs  6-18 years | 0.625 | 0.53 |
| Squamous epithelial cells | 3-5 years vs  >18 years | 0.082 | 0.93 |
| Squamous epithelial cells | 6-18 years vs  >18 years | 0.860 | 0.39 |
| WBC & >Bacteria none seen | 3 months-2 years vs  3-5 years | 0.324 | 0.75 |
| WBC & >Bacteria none seen | 3 months-2 years vs  6-18 years | 0.823 | 0.41 |
| WBC & >Bacteria none seen | 3 months-2 years vs  >18 years | 0.598 | 0.55 |
| WBC & >Bacteria none seen | 3-5 years vs  6-18 years | 0.781 | 0.43 |
| WBC & >Bacteria none seen | 3-5 years vs  >18 years | 0.329 | 0.74 |
| WBC & >Bacteria none seen | 6-18 years vs  >18 years | 0.727 | 0.47 |
| WBC & >Bacteria few | 3 months-2 years vs  3-5 years | 0.305 | 0.76 |
| WBC & >Bacteria few | 3 months-2 years vs  6-18 years | 0.971 | 0.33 |
| WBC & >Bacteria few | 3 months-2 years vs  >18 years | 1.441 | 0.15 |
| WBC & >Bacteria few | 3-5 years vs  6-18 years | 2.030 | 0.04 |
| WBC & >Bacteria few | 3-5 years vs  >18 years | 1.973 | 0.05 |
| WBC & >Bacteria few | 6-18 years vs  >18 years | 0.991 | 0.32 |
| WBC & >Bacteria moderate | 3 months-2 years vs  3-5 years | 1.073 | 0.28 |
| WBC & >Bacteria moderate | 3 months-2 years vs  6-18 years | 0.280 | 0.78 |
| WBC & >Bacteria moderate | 3 months-2 years vs  >18 years | 0.846 | 0.40 |
| WBC & >Bacteria moderate | 3-5 years vs  6-18 years | 0.950 | 0.35 |
| WBC & >Bacteria moderate | 3-5 years vs  >18 years | 1.373 | 0.17 |
| WBC & >Bacteria moderate | 6-18 years vs  >18 years | 0.198 | 0.84 |
| WBC & >Bacteria many | 3 months-2 years vs  3-5 years | 1.530 | 0.12 |
| WBC & >Bacteria many | 3 months-2 years vs  6-18 years | 0.801 | 0.42 |
| WBC & >Bacteria many | 3 months-2 years vs  >18 years | 0.600 | 0.55 |
| WBC & >Bacteria many | 3-5 years vs  6-18 years | 0.530 | 0.60 |
| WBC & >Bacteria many | 3-5 years vs  >18 years | 0.496 | 0.62 |
| WBC & >Bacteria many | 6-18 years vs  >18 years | 0.333 | 0.74 |
| WBC & Squamous epithelial cells ≥ none seen | 3 months-2 years vs  3-5 years | 0.279 | 0.78 |
| WBC & Squamous epithelial cells ≥ none seen | 3 months-2 years vs  6-18 years | 0.715 | 0.47 |
| WBC & Squamous epithelial cells ≥ none seen | 3 months-2 years vs  >18 years | 0.499 | 0.62 |
| WBC & Squamous epithelial cells ≥ none seen | 3-5 years vs  6-18 years | 0.781 | 0.43 |
| WBC & Squamous epithelial cells ≥ none seen | 3-5 years vs  >18 years | 0.329 | 0.74 |
| WBC & Squamous epithelial cells ≥ none seen | 6-18 years vs  >18 years | 0.73 | 0.47 |
| WBC & Squamous epithelial cells ≤ 30 | 3 months-2 years vs  3-5 years | 0.244 | 0.81 |
| WBC & Squamous epithelial cells ≤ 30 | 3 months-2 years vs  6-18 years | 1.599 | 0.11 |
| WBC & Squamous epithelial cells ≤ 30 | 3 months-2 years vs  >18 years | 1.572 | 0.12 |
| WBC & Squamous epithelial cells ≤ 30 | 3-5 years vs  6-18 years | 2.134 | 0.03 |
| WBC & Squamous epithelial cells ≤ 30 | 3-5 years vs  >18 years | 2.63 | 0.009 |
| WBC & Squamous epithelial cells ≤ 30 | 6-18 years vs  >18 years | 0.595 | 0.55 |
| WBC & Squamous epithelial cells ≤ 15 | 3 months-2 years vs  3-5 years | 0.140 | 0.89 |
| WBC & Squamous epithelial cells ≤ 15 | 3 months-2 years vs  6-18 years | 2.665 | 0.008 |
| WBC & Squamous epithelial cells ≤ 15 | 3 months-2 years vs  >18 years | 2.914 | 0.004 |
| WBC & Squamous epithelial cells ≤ 15 | 3-5 years vs  6-18 years | 3.956 | 0.0001 |
| WBC & Squamous epithelial cells ≤ 15 | 3-5 years vs  >18 years | 4.932 | <0.0001 |
| WBC & Squamous epithelial cells ≤ 15 | 6-18 years vs  >18 years | 1.057 | 0.29 |
| WBC & Squamous epithelial cells ≤ 3 | 3 months-2 years vs  3-5 years | 1.082 | 0.28 |
| WBC & Squamous epithelial cells ≤ 3 | 3 months-2 years vs  6-18 years | 4.390 | <0.0001 |
| WBC & Squamous epithelial cells ≤ 3 | 3 months-2 years vs  >18 years | 3.910 | 0.0001 |
| WBC & Squamous epithelial cells ≤ 3 | 3-5 years vs  6-18 years | 5.102 | <0.0001 |
| WBC & Squamous epithelial cells ≤ 3 | 3-5 years vs  >18 years | 7.070 | <0.0001 |
| WBC & Squamous epithelial cells ≤ 3 | 6-18 years vs  >18 years | 0.793 | 0.43 |
| WBC & Squamous epithelial cells = none seen | 3 months-2 years vs  3-5 years | 1.940 | 0.052 |
| WBC & Squamous epithelial cells = none seen | 3 months-2 years vs  6-18 years | 4.562 | <0.0001 |
| WBC & Squamous epithelial cells = none seen | 3 months-2 years vs  >18 years | 3.195 | 0.001 |
| WBC & Squamous epithelial cells = none seen | 3-5 years vs  6-18 years | 4.300 | <0.0001 |
| WBC & Squamous epithelial cells = none seen | 3-5 years vs  >18 years | 6.854 | <0.0001 |
| WBC & Squamous epithelial cells = none seen | 6-18 years vs  >18 years | 0.266 | 0.80 |
| Leukocyte esterase > - & Nitrite - or + | 3 months-2 years vs  3-5 years | 1.714 | 0.087 |
| Leukocyte esterase > - & Nitrite - or + | 3 months-2 years vs  6-18 years | 2.473 | 0.013 |
| Leukocyte esterase > - & Nitrite - or + | 3 months-2 years vs  >18 years | 0.997 | 0.32 |
| Leukocyte esterase > - & Nitrite - or + | 3-5 years vs  6-18 years | 1.249 | 0.21 |
| Leukocyte esterase > - & Nitrite - or + | 3-5 years vs  >18 years | 0.081 | 0.94 |
| Leukocyte esterase > - & Nitrite - or + | 6-18 years vs  >18 years | 1.652 | 0.10 |
| Leukocyte esterase > - & Nitrite + | 3 months-2 years vs  3-5 years | 3.091 | 0.002 |
| Leukocyte esterase > - & Nitrite + | 3 months-2 years vs  6-18 years | 2.690 | 0.007 |
| Leukocyte esterase > - & Nitrite + | 3 months-2 years vs  >18 years | 3.238 | 0.001 |
| Leukocyte esterase > - & Nitrite + | 3-5 years vs  6-18 years | 0.174 | 0.86 |
| Leukocyte esterase > - & Nitrite + | 3-5 years vs  >18 years | 0.464 | 0.64 |
| Leukocyte esterase > - & Nitrite + | 6-18 years vs  >18 years | 0.465 | 0.64 |

Supplemental Table 6 Legend

Abbreviations:

ED= Emergency department

WBC=White blood cell

Supplemental Table 7. Urinalysis Microscopic Results’ Sensitivity & Specificity Analysis: WBC Combined with Bacteria (n=80,949)

| WBC (WBCs/HPF) &  >Bacteria none seen (HPF)  count on urine microscope  **ROC Curve (AUC=0.722)** | Sensitivity for detecting a uropathogen  n=28,993 | Specificity for detecting a uropathogen  n=51,956 | % of ordered  urine cultures that would be  processed  n=80,949 | % of uropathogens that would be  excluded from culture  n=28,993 | % of non-uropathogens that would be  excluded from culture  n=2,960 |
| --- | --- | --- | --- | --- | --- |
| WBC >0 & >Bacteria none seen | 100% (28,993) | 0% (0) | 100% (80,949) | 0% (0) | 0% (0) |
| WBC >3 & >Bacteria none seen | 99% (28,738) | 2% (1,254) | 98% (79,440) | 1% (255) | 0.1% (23) |
| WBC >10 & >Bacteria none seen | 81% (23,581) | 48% (24,964) | 62% (50,573) | 19% (5,4812 | 32% (947) |
| WBC >30 & >Bacteria none seen | 64% (18,567) | 72% (37,378) | 41% (33,145) | 36% (10,426) | 57% (1,673) |
| WBC >50 & >Bacteria none seen | 47% (13,522) | 85% (44,333) | 26% (21,145) | 53% (15,471) | 72% (2,160) |
| WBC >50 & >Bacteria none seen | 38% (11,093) | 90% (46,554) | 20% (16,495) | 62% (17,900) | 80% (2,363) |

| WBC (WBCs/HPF) &  > Bacteria few (HPF)  count on urine microscope  **ROC Curve (AUC=0.711)** | Sensitivity for detecting a uropathogen  n=28,993 | Specificity for detecting a uropathogen  n=51,956 | % of ordered  urine cultures that would be  processed  n=80,949 | % of uropathogens that would be  excluded from culture  n=28,993 | % of non-uropathogens that would be  excluded from culture  n=2,960 |
| --- | --- | --- | --- | --- | --- |
| WBC >0 & >Bacteria few | 80% (23,162) | 42% (21,652) | 66% (53,466) | 20% (5,831) | 28% (827) |
| WBC >3 & > Bacteria few | 80% (23,097) | 42% (21,918) | 66% (53,135) | 20% (5,896) | 28% (835) |
| WBC >10 & > Bacteria few | 71% (20,621) | 61% (31,734) | 51% (40,843) | 29% (8,372) | 45% 1,333) |
| WBC >30 & > Bacteria few | 58% (16,805) | 77% (40,279) | 35% (28,482) | 42% (12,188) | 64% (1,884) |
| WBC >50 & > Bacteria few | 43% (12,514) | 88% (45,526) | 23% (18,944) | 57% (16,479) | 78% (2,286) |
| WBC >50 & > Bacteria few | 36% (10,320) | 91% (47,331) | 18% (14,954) | 64% (18,673) | 83% (2,458) |

| WBC (WBCs/HPF) &  >Bacteria moderate (HPF)  count on urine microscope  **ROC Curve (AUC=0.634)** | Sensitivity for detecting a uropathogen  n=28,993 | Specificity for detecting a uropathogen  n=51,956 | % of ordered  urine cultures that would be  processed  n=80,949 | % of uropathogens that would be  excluded from culture  n=28,993 | % of non-uropathogens that would be  excluded from culture  n=2,960 |
| --- | --- | --- | --- | --- | --- |
| WBC >0 & > Bacteria moderate | 38% (10,926) | 87% (45,503) | 22% (17,379) | 62% (18,067) | 80% (2,364) |
| WBC >3 & >Bacteria moderate | 38% (10,902) | 87% (45,527) | 22% (17,331) | 62% (18,091) | 80% (2,365) |
| WBC >10 & >Bacteria moderate | 36% (10,280) | 90% (46,601) | 19% (15,635) | 64% (18,713) | 83% (2,461) |
| WBC >30 & >Bacteria moderate | 31% (8,884) | 93% (48,179) | 16% (12,661) | 69% (20,109) | 89% (2,20) |
| WBC >50 & >Bacteria moderate | 24% (7,022) | 95% (49,447) | 12% (9,531) | 76% (22,971) | 92% (2,725) |
| WBC >50 & >Bacteria moderate | 21% (5,943) | 97% (50,053) | 10% (7,846) | 79% (23,050) | 94% (2,778) |

| WBC (WBCs/HPF) &  >Bacteria many (HPF)  count on urine microscope  **ROC Curve (AUC=0.587)** | Sensitivity for detecting a uropathogen  n=28,993 | Specificity for detecting a uropathogen  n=51,956 | % of ordered  urine cultures that would be  processed  n=80,949 | % of uropathogens that would be  excluded from culture  n=28,993 | % of non-uropathogens that would be  excluded from culture  n=2,960 |
| --- | --- | --- | --- | --- | --- |
| WBC >0 & > Bacteria many | 22% (6,277) | 96% (49,656) | 11% (8,577) | 78% (22,716) | 94% (2,768) |
| WBC >3 & > Bacteria many | 22% (6,263) | 96% (49,668) | 11% (8,551) | 78% (22,730) | 94% (2,768) |
| WBC >10 & > Bacteria many | 21% (5,946) | 96% (49,893) | 10% (8,009) | 79% (23,047) | 94% (2,791) |
| WBC >30 & > Bacteria many | 18% (5,153) | 97% (50,347) | 8% (6,762) | 82% (23,840) | 96% (2,831) |
| WBC >50 & > Bacteria many | 14% (4,132) | 98% (50,811) | 7% (5,277) | 86% (24,861) | 97% (2,871) |
| WBC >50 & > Bacteria many | 12% (3,545) | 98% (51,042) | 6% (4,459) | 88% (25,448) | 98% (2,888) |

Supplemental Table 8. Urinalysis Microscopic Results’ Sensitivity & Specificity Analysis: WBC Combined with Squamous Epithelial Cells (n=80,949)

| WBC (WBCs/HPF) &  Squamous epithelial cells $\geq0$(LPF)  count on urine microscope  **ROC Curve (AUC=0.722)** | Sensitivity for detecting a uropathogen  n=28,993 | Specificity for detecting a uropathogen  n=51,956 | % of ordered  urine cultures that would be  processed  n=80,949 | % of uropathogens that would be  excluded from culture  n=28,993 | % of non-uropathogens that would be  excluded from culture  n=2,960 |
| --- | --- | --- | --- | --- | --- |
| WBC >0 & Squamous epithelial cells $\geq0$ | 100% (28,993) | 0% (0) | 100% (80,949) | 0% (0) | 0% (0) |
| WBC >3 & Squamous epithelial cells$\geq0$ | 99% (28,738) | 2% (1,254) | 98% (79,440) | 1% (255) | 1% (23) |
| WBC >10 & Squamous epithelial cells $\geq0$ | 81% (23,581) | 48% (24,964) | 63% (50,573) | 19% (5,412) | 32% (947) |
| WBC >30 & Squamous epithelial cells $\geq0$ | 64% (18,567) | 72% (37,378) | 41% (33,145) | 36% (10,426) | 57% (1,673) |
| WBC >50 & Squamous epithelial cells$\geq0$ | 47% (13,522) | 85% (44,333) | 26% (21,145) | 53% (15,471) | 73% (2,160) |
| WBC >50 & Squamous epithelial cells $\geq0$ | 38% (11,093) | 90% (46,554) | 20% (16,495) | 62% (17,900) | 80% (2,363) |

| WBC (WBCs/HPF) &  Squamous epithelial cells $\leq30$ (LPF)  count on urine microscope  **ROC Curve (AUC=0.688)** | Sensitivity for detecting a uropathogen  n=28,993 | Specificity for detecting a uropathogen  n=51,956 | % of ordered  urine cultures that would be  processed  n=80,949 | % of uropathogens that would be  excluded from culture  n=28,993 | % of non-uropathogens that would be  excluded from culture  n=2,960 |
| --- | --- | --- | --- | --- | --- |
| WBC >0 & Squamous epithelial cells $\leq30$ | 65% (18,943) | 37% (19,318) | 64% (51,581) | 35% (10,050) | 46% (1,360) |
| WBC >3 & Squamous epithelial cells $\leq30$ | 65% (18,746) | 39% (20,400) | 62% (50,302) | 35% (10,247) | 47% (1,378) |
| WBC >10 & Squamous epithelial cells $\leq30$ | 52% (15,108) | 72% (37,563) | 37% (29,501) | 48% (13,885) | 64% (1,898) |
| WBC >30 & Squamous epithelial cells $\leq30$ | 42% (12,145) | 85% (43,982) | 25% (20,119) | 58% (16,848) | 74% (2,179) |
| WBC >50 & Squamous epithelial cells $\leq30$ | 31% (9,061) | 91% (47,453) | 17% (13,564) | 69% (19,932) | 82% (2,422) |
| WBC >50 & Squamous epithelial cells $\leq30$ | 26% (7,507) | 94% (48,633) | 13% (10,830) | 74% (21,486) | 86% (2,538) |

| WBC (WBCs/HPF) &  Squamous epithelial cells $\leq15$ (LPF)  count on urine microscope  **ROC Curve (AUC=0.666)** | Sensitivity for detecting a uropathogen  n=28,993 | Specificity for detecting a uropathogen  n=51,956 | % of ordered  urine cultures that would be  processed  n=80,949 | % of uropathogens that would be  excluded from culture  n=28,993 | % of non-uropathogens that would be  excluded from culture  n=2,960 |
| --- | --- | --- | --- | --- | --- |
| WBC >0 & Squamous epithelial cells $\leq15$ | 50% (14,603) | 50% (25,871) | 50% (40,688) | 50% (14,390) | 57% (1,698) |
| WBC >3 & Squamous epithelial cells $\leq15$ | 50% (14,434) | 52% (26,849) | 49% (39,541) | 50% (14,559) | 58% (1,714) |
| WBC >10 & Squamous epithelial cells $\leq15$ | 40% (11,605) | 79% (41,096) | 28% (22,465) | 60% (17,388) | 71% (2.109) |
| WBC >30 & Squamous epithelial cells $\leq15$ | 33% (9,467) | 88% (45,817) | 19% (15,606) | 67% (19,526) | 78% (2,309) |
| WBC >50 & Squamous epithelial cells $\leq15$ | 25% (7,131) | 93% (48,419) | 13% (10,668) | 75% (21,862) | 84% (2,500) |
| WBC >50 & Squamous epithelial cells $\leq15$ | 21% (5,969) | 95% (49,310) | 11% (8,615) | 79% (23,024) | 88% (2,594) |

| WBC (WBCs/HPF) &  & Squamous epithelial cells $\leq3$ (LPF)  count on urine microscope  **ROC Curve (AUC=0.612)** | Sensitivity for detecting a uropathogen  n=28,993 | Specificity for detecting a uropathogen  n=51,956 | % of ordered  urine cultures that would be  processed  n=80,949 | % of uropathogens that would be  excluded from culture  n=28,993 | % of non-uropathogens that would be  excluded from culture  n=2,960 |
| --- | --- | --- | --- | --- | --- |
| WBC >0 & Squamous epithelial cells $\leq3$ | 27% (7,842) | 70% (36,333) | 29% (23,465) | 73% (21,151) | 75% (2,223) |
| WBC >3 & Squamous epithelial cells $\leq3$ | 27% (7,712) | 71% (37,072) | 28% (22,596) | 73% (21,281) | 75% (2,223) |
| WBC >10 & Squamous epithelial cells $\leq3$ | 21% (6,180) | 89% (45,985) | 15% (12,151) | 79% (22,813) | 82% (2,423) |
| WBC >30 & Squamous epithelial cells $\leq3$ | 18% (5,174) | 93% (48,454) | 11% (8,676) | 82% (23,819) | 86% (2,537) |
| WBC >50 & Squamous epithelial cells $\leq3$ | 14% (3,946) | 96% (49,896) | 7% (6,006) | 87% (25,047) | 90% (2,665) |
| WBC >50 & Squamous epithelial cells $\leq3$ | 11% (3,312) | 97% (50,386) | 6% (4,882) | 89% (25,681) | 92% (2,722) |

| WBC (WBCs/HPF) &  & Squamous epithelial cells $\leq none seen$ (LPF)  count on urine microscope  **ROC Curve (AUC=0.579)** | Sensitivity for detecting a uropathogen  n=28,993 | Specificity for detecting a uropathogen  n=51,956 | % of ordered  urine cultures that would be  processed  n=80,949 | % of uropathogens that would be  excluded from culture  n=28,993 | % of non-uropathogens that would be  excluded from culture  n=2,960 |
| --- | --- | --- | --- | --- | --- |
| WBC >0 & Squamous epithelial cells $=0$ | 17% (4,870) | 81% (42,124) | 18% (14,702) | 83% (24,123) | 84% (2,485) |
| WBC >3 & Squamous epithelial cells $=0$ | 16% (4,773) | 82% (42,686) | 17% (14,043) | 84% (24,220) | 84% (2,492) |
| WBC >10 & Squamous epithelial cells $=0$ | 14% (3,945) | 93% (48,303) | 9% (7,598) | 86% (25,048) | 88% (2,607) |
| WBC >30 & Squamous epithelial cells $=0$ | 12% (3,409) | 96% (49,721) | 7% (5,644) | 88% (25,584) | 90% (2,669) |
| WBC >50 & Squamous epithelial cells $=0$ | 9% (2,702) | 97% (50,579) | 5% (4,079) | 91% (26,291) | 93% (2,743) |
| WBC >50 & Squamous epithelial cells $=0$ | 8% (2,344) | 98% (50,881) | 4% (3,419) | 92% (26,649) | 94% (2,781) |

Supplemental Table 9. Urinalysis Microscopic Results’ Sensitivity & Specificity Analysis Leukocyte Esterase Combined with Nitrite (n=80,949)

| Urinalysis Leukocyte esterase &  Urinalysis Nitrite – or +  value on urine macroscopic examination  **ROC Curve (AUC=0.695)** | Sensitivity for detecting a uropathogen  n=28,993 | Specificity for detecting a uropathogen  n=51,956 | % of ordered  urine cultures that would be  processed  n=80,949 | % of uropathogens that would be  excluded from culture  n=28,993 | % of non-uropathogens that would be  excluded from culture  n=2,960 |
| --- | --- | --- | --- | --- | --- |
| Leukocyte esterase > - & Nitrite - or + | 100% (28,993) | 0% (0) | 100% (80,949) | 0% (0) | 0% (0) |
| Leukocyte esterase > trace & Nitrite - or + | 82% (23,645) | 46% (23,660) | 64% (51,941) | 18% (5,348) | 29% (872) |
| Leukocyte esterase > small & Nitrite - or + | 73% (21,071) | 58% (29,761) | 53% (43,266) | 27% (7,922) | 40% (1,192) |
| Leukocyte esterase > moderate & Nitrite - or + | 60% (17,268) | 71% (37,000) | 40% (32,224) | 41% (11,725) | 56% (1,645) |
| Leukocyte esterase > moderate & Nitrite - or + | 43% (12,326) | 84% (43,546) | 26% (20,736) | 58% (16,667) | 72% (2,118) |

| Urinalysis Leukocyte esterase &  Urinalysis Nitrite +  value on urine macroscopic examination  **ROC Curve (AUC=0.628)** | Sensitivity for detecting a uropathogen  n=28,993 | Specificity for detecting a uropathogen  n=51,956 | % of ordered  urine cultures that would be  processed  n=80,949 | % of uropathogens that would be  excluded from culture  n=28,993 | % of non-uropathogens that would be  excluded from culture  n=2,960 |
| --- | --- | --- | --- | --- | --- |
| Leukocyte esterase > - & Nitrite + | 29% (8,522) | 96% (49,916) | 13% (10,562) | 71% (20,471) | 94% (2,775) |
| Leukocyte esterase > - & Nitrite + | 27% (7,725) | 98% (50,275) | 12% (9,406) | 73% (21,268) | 95% (2,803) |
| Leukocyte esterase > trace & Nitrite + | 25% (7,201) | 97% (50,387) | 11% (8,770) | 75% (21,792) | 95% (2,816) |
| Leukocyte esterase > small & Nitrite + | 21% (6,206) | 98% (50,642) | 9% (7,520) | 79% (22,787) | 96% (2,845) |
| Leukocyte esterase > moderate & Nitrite + | 16% (4,730) | 98% (51,003) | 7% (5,683) | 84% (24,263) | 97% (2,880) |

Supplemental Table 10. **Emergency Department Population**: Urinalysis Microscopic Results’ Sensitivity & Specificity Analysis WBC Combined with Bacteria (n=20,716):

| WBC (WBCs/HPF) &  >Bacteria none seen (HPF)  count on urine microscope  **ROC Curve (AUC=0.727)** | Sensitivity for detecting a uropathogen  n=7,631 | Specificity for detecting a uropathogen  n=13,085 | % of ordered  urine cultures that would be  processed  n=20,716 | % of uropathogens that would be  excluded from culture  n=7,631 | % of non-uropathogens that would be  excluded from culture  n=668 |
| --- | --- | --- | --- | --- | --- |
| WBC >0 & >Bacteria none seen | 100% (7,631) | 0% (0) | 100% (7,756) | 0% (0) | 0% (0) |
| WBC >3 & >Bacteria none seen | 99% (7,562) | 3% (352) | 98% (20,295) | 1% (69) | 0.6% (4) |
| WBC >10 & >Bacteria none seen | 83% (6,321) | 46% (6,072) | 64% (13,334) | 17% (1,310) | 35% (232) |
| WBC >30 & >Bacteria none seen | 67% (5,101) | 70% (9,125) | 44% (9,061) | 33% (2,530) | 57% (383) |
| WBC >50 & >Bacteria none seen | 50% (3,838) | 84% (10,968) | 29% (5,955) | 50% (3,793) | 73% (487) |
| WBC >50 & >Bacteria none seen | 42% (3,197) | 88% (11,563) | 23% (4,719) | 58% (4,437) | 80% (533) |

| WBC (WBCs/HPF) &  > few bacteria (HPF)  count on urine microscope  **ROC Curve (AUC=0.718)** | Sensitivity for detecting a uropathogen  n=7,631 | Specificity for detecting a uropathogen  n=13,085 | % of ordered  urine cultures that would be  processed  n=20,716 | % of uropathogens would be  excluded from culture  n=7,631 | % of non-uropathogens would be  excluded from culture  n=668 |
| --- | --- | --- | --- | --- | --- |
| WBC >0 & >Bacteria few | 80% (6,091) | 42% (5,508) | 66% (13,668) | 20% (1,540) | 29% (198) |
| WBC >3 & > Bacteria few | 80% (6,073) | 42% (5,577) | 66% (13,581) | 20% (1,558) | 30% (199) |
| WBC >10 & > Bacteria few | 72% (5,503) | 61% (7,941) | 52% (10,647) | 28% (2,128) | 47% (316) |
| WBC >30 & > Bacteria few | 60% (4,570) | 76% (10,018) | 37% (7,637) | 40% (3,061) | 65% (435) |
| WBC >50 & > Bacteria few | 46% (3,507) | 87% (11,354) | 25% (5,238) | 54% (4,124) | 77% (516) |
| WBC >50 & > Bacteria few | 39% (2,940) | 90% (11,833) | 20% (4,192) | 62% (4,691) | 83% (556) |

| WBC (WBCs/HPF) &  >Bacteria moderate (HPF)  count on urine microscope  **ROC Curve (AUC=0.637)** | Sensitivity for detecting a uropathogen  n=7,631 | Specificity for detecting a uropathogen  n=13,085 | % of ordered  urine cultures that would be  processed  n=20,716 | % of uropathogens would be  excluded from culture  n=7,631 | % of non-uropathogens would be  excluded from culture  n=668 |
| --- | --- | --- | --- | --- | --- |
| WBC >0 & > Bacteria moderate | 40% (3,029) | 86% (11,231) | 24% (4,883) | 60% (4,602) | 75% (500) |
| WBC >3 & >Bacteria moderate | 40% (3,021) | 86% (11,236) | 24% (4,970) | 60% (4,610) | 75% (500) |
| WBC >10 & >Bacteria moderate | 38% (2,851) | 88% (11,544) | 21% (4,392) | 62% (4,780) | 80% (531) |
| WBC >30 & >Bacteria moderate | 32% (2,468) | 92% (11,982) | 17% (3,571) | 68% (5,163) | 86% (571) |
| WBC >50 & >Bacteria moderate | 26% (1,995) | 94% (12,355) | 13% (2,725) | 74% (5,636) | 91% (604) |
| WBC >50 & >Bacteria moderate | 22% (1,702) | 96% (12,540) | 11% (2,247) | 78% (5,929) | 93% (623) |

| WBC (WBCs/HPF) &  Bacteria (HPF)  count on urine microscope  **ROC Curve (AUC=0.591)** | Sensitivity for detecting a uropathogen  n=7,631 | Specificity for detecting a uropathogen  n=13,085 | % of ordered  urine cultures that would be  processed  n=20,716 | % of uropathogens would be  excluded from culture  n=7,631 | % of non-uropathogens would be  excluded from culture  n=668 |
| --- | --- | --- | --- | --- | --- |
| WBC >0 & > Bacteria many | 23% (1,745) | 95% (12,450) | 12% (2,380) | 77% (5,886) | 92% (614) |
| WBC >3 & > Bacteria many | 23% (1,739) | 95% (12,453) | 11% (2,371) | 77% (5,892) | 92% (614) |
| WBC >10 & > Bacteria many | 22% (1,655) | 96% (12,514) | 11% (2,226) | 78% (5,976) | 93% (621) |
| WBC >30 & > Bacteria many | 19% (1,440) | 97% (12,638) | 9% (1,887) | 81% (6,191) | 95% (630) |
| WBC >50 & > Bacteria many | 15% (1,173) | 98% (12,765) | 7% (1,493) | 85% (6,458) | 96% (641) |
| WBC >50 & > Bacteria many | 13% (1,004) | 98% (12,835) | 6% (1,254) | 87% (6,627) | 97% (647) |

Supplemental Table 11. **Emergency Department Population** Urinalysis Microscopic Results’ Sensitivity & Specificity Analysis WBC Combined with Squamous Epithelial Cells (n=20,716)

| WBC (WBCs/HPF) &  Squamous epithelial cells $\geq0$(LPF)  count on urine microscope  **ROC Curve (AUC=0.727)** | Sensitivity for detecting a uropathogen  n=7,631 | Specificity for detecting a uropathogen  n=13,085 | % of ordered  urine cultures that would be  processed  n=20,716 | % of uropathogens would be  excluded from culture  n=7,631 | % of non-uropathogens would be  excluded from culture  n=668 |
| --- | --- | --- | --- | --- | --- |
| WBC >0 & Squamous epithelial cells $\geq0$ | 100% (20,716) | 0% (0) | 100% (20,716) | 0% (0) | 0% (0) |
| WBC >3 & Squamous epithelial cells$\geq0$ | 99% (7,562) | 3% (352) | 98% (20,295) | 1% (69) | 1% (4) |
| WBC >10 & Squamous epithelial cells $\geq0$ | 83% (6,321) | 46% (6,072) | 64% (13,334) | 17% (1,310) | 35% (232) |
| WBC >30 & Squamous epithelial cells $\geq0$ | 67% (5,101) | 70% (9,125) | 44% (9,061) | 33% (2,530) | 57% (383) |
| WBC >50 & Squamous epithelial cells$\geq0$ | 50% (3,838) | 84% (10,968) | 29% (5,955) | 50% (3,793) | 73% (487) |
| WBC >50 & Squamous epithelial cells $\geq0$ | 42% (3,197) | 88% (11,583) | 23% (4,719) | 58% (4,434) | 80% (533) |

| WBC (WBCs/HPF) &  Squamous epithelial cells $\leq30$ (LPF)  count on urine microscope  **ROC Curve (AUC=0.695)** | Sensitivity for detecting a uropathogen  n=7,631 | Specificity for detecting a uropathogen  n=13,085 | % of ordered  urine cultures that would be  processed  n=20,716 | % of uropathogens would be  excluded from culture  n=7,631 | % of non-uropathogens would be  excluded from culture  n=668 |
| --- | --- | --- | --- | --- | --- |
| WBC >0 & Squamous epithelial cells $\leq30$ | 65% (8,520) | 33% (2,533) | 66% (13,618) | 35% (4,565) | 46% (306) |
| WBC >3 & Squamous epithelial cells $\leq30$ | 63% (8,204) | 34% (2,587) | 64% (13,248) | 37% (4,881) | 47% (310) |
| WBC >10 & Squamous epithelial cells $\leq30$ | 30% (3,961) | 46% (3,501) | 39% (8,091) | 70% (9,124) | 65% (435) |
| WBC >30 & Squamous epithelial cells $\leq30$ | 18% (2,286) | 55% (4,205) | 28% (5,712) | 83% (10,799) | 74% (495) |
| WBC >50 & Squamous epithelial cells $\leq30$ | 10% (1,338) | 65% (4,962) | 19% (4,007) | 90% (11,747) | 81% (544) |
| WBC >50 & Squamous epithelial cells $\leq30$ | 8% (1,000) | 71% (5,399) | 16% (3,232) | 92% (12,085) | 85% (568) |

| WBC (WBCs/HPF) &  Squamous epithelial cells $\leq15$ (LPF)  count on urine microscope  **ROC Curve (AUC=0.678)** | Sensitivity for detecting a uropathogen  n=7,631 | Specificity for detecting a uropathogen  n=13,085 | % of ordered  urine cultures that would be  processed  n=20,716 | % of uropathogens would be  excluded from culture  n=7,631 | % of non-uropathogens would be  excluded from culture  n=668 |
| --- | --- | --- | --- | --- | --- |
| WBC >0 & Squamous epithelial cells $\leq15$ | 53% (4,046) | 47% (6,168) | 53% (10,963) | 47% (3,585) | 55% (370) |
| WBC >3 & Squamous epithelial cells $\leq15$ | 53% (3,999) | 49% (6,454) | 51% (10,630) | 48% (3,632) | 56% (374) |
| WBC >10 & Squamous epithelial cells $\leq15$ | 43% (3,298) | 77% (10,053) | 31% (6,331) | 57% (4,332) | 72% (480) |
| WBC >30 & Squamous epithelial cells $\leq15$ | 37% (2,790) | 86% (11,281) | 22% (4,594) | 63% (4,841) | 77% (517) |
| WBC >50 & Squamous epithelial cells $\leq15$ | 29% (2,182) | 92% (11,999) | 16% (3,268) | 71% (5,449) | 83% (557) |
| WBC >50 & Squamous epithelial cells $\leq15$ | 24% (1,838) | 94% (12,267) | 13% (2,656) | 76% (5,793) | 87% (580) |
| WBC (WBCs/HPF) &  & Squamous epithelial cells $\leq3$ (LPF)  count on urine microscope  **ROC Curve (AUC=0.623)** | Sensitivity for detecting a uropathogen  n=7,631 | Specificity for detecting a uropathogen  n=13,085 | % of ordered  urine cultures that would be  processed  n=20,716 | % of uropathogens would be  excluded from culture  n=7,631 | % of non-uropathogens would be  excluded from culture  n=668 |
| WBC >0 & Squamous epithelial cells $\leq3$ | 30% (2,315) | 68% (8,833) | 32% (6,567) | 70% (5,316) | 73% (488) |
| WBC >3 & Squamous epithelial cells $\leq3$ | 30% (2,277) | 69% (9,062) | 30% (6,300) | 70% (5,354) | 74% (492) |
| WBC >10 & Squamous epithelial cells $\leq3$ | 24% (1,867) | 87% (11,352) | 17% (3,600) | 76% (5,764) | 82% (550) |
| WBC >30 & Squamous epithelial cells $\leq3$ | 21% (1,614) | 92% (12,024) | 13% (2,675) | 78% (6,017) | 85% (568) |
| WBC >50 & Squamous epithelial cells $\leq3$ | 17% (1,277) | 95% (12,422) | 9% (1,940) | 83% (6,354) | 89% (594) |
| WBC >50 & Squamous epithelial cells $\leq3$ | 14% (1,074) | 96% (12,575) | 8% (1,584) | 86% (6,557) | 92% (609) |

| WBC (WBCs/HPF) &  & Squamous epithelial cells $=0$(LPF)  count on urine microscope  **ROC Curve (AUC=0.587)** | Sensitivity for detecting a uropathogen  n=7,631 | Specificity for detecting a uropathogen  n=13,085 | % of ordered  urine cultures that would be  processed  n=20,716 | % of uropathogens would be  excluded from culture  n=7,631 | % of non-uropathogens would be  excluded from culture  n=668 |
| --- | --- | --- | --- | --- | --- |
| WBC >0 & Squamous epithelial cells $=0$ | 19% (1,435) | 80% (10,457) | 20% (4,063) | 81% (6,196) | 82% (546) |
| WBC >3 & Squamous epithelial cells $=0$ | 18% (1,405) | 81% (10,637) | 19% (3,853) | 82% (6,226) | 82% (550) |
| WBC >10 & Squamous epithelial cells $=0$ | 15% (1,178) | 92% (12,070) | 11% (2,193) | 85% (6,453) | 88% (588) |
| WBC >30 & Squamous epithelial cells $=0$ | 14% (1,050) | 95% (12,440) | 8% (1,695) | 86% (6,581) | 90% (600) |
| WBC >50 & Squamous epithelial cells $=0$ | 11% (866) | 97% (12,660) | 6% (1,291) | 89% (6,765) | 92% (613) |
| WBC >50 & Squamous epithelial cells $=0$ | 10% (766) | 97% (12,744) | 5% (1,107) | 90% (6,865) | 94% (626) |

Supplemental Table 12. **Emergency Department Population** Urinalysis Microscopic Results’ Sensitivity & Specificity Analysis Leukocyte Esterase Combined with Nitrite (n=20,716)

| Urinalysis Leukocyte esterase &  Urinalysis Nitrite - or +  value on urine macroscopic examination  **ROC Curve (AUC=0.702)** | Sensitivity for detecting a uropathogen  n=7,631 | Specificity for detecting a uropathogen  n=13,085 | % of ordered  urine cultures that would be  processed  n=20,716 | % of uropathogens would be  excluded from culture  n=7,631 | % of non-uropathogens would be  excluded from culture  n=668 |
| --- | --- | --- | --- | --- | --- |
| Leukocyte esterase > - & Nitrite - or + | 100% (7,631) | 0% (0) | 100% (20,716) | 0% (0) | 0% (0) |
| Leukocyte esterase > trace & Nitrite - or + | 82% (6,278) | 45% (5,867) | 18% (1,353) | 65% (13,496) | 30% (203) |
| Leukocyte esterase > small & Nitrite - or + | 75% (5,705) | 57% (7,396) | 55% (11,394) | 25% (1,926) | 42% (279) |
| Leukocyte esterase > moderate & Nitrite - or + | 62% (4,729) | 71% (9,217) | 41% (8,597) | 38% (2,902) | 56% (372) |
| Leukocyte esterase > moderate & Nitrite - or + | 45% (3,438) | 83% (10,890) | 27% (5,633) | 55% (4,193) | 72% (483) |

| Urinalysis Leukocyte esterase &  Urinalysis Nitrite +  value on urine macroscopic examination  **ROC Curve (AUC=0.627)** | Sensitivity for detecting a uropathogen  n=7,631 | Specificity for detecting a uropathogen  n=13,085 | % of ordered  urine cultures that would be  processed  n=20,716 | % of uropathogens would be  excluded from culture  n=7,631 | % of non-uropathogens would be  excluded from culture  n=668 |
| --- | --- | --- | --- | --- | --- |
| Leukocyte esterase > - & Nitrite + | 30% (2,264) | 96% (12,511) | 14% (2,838) | 70% (5,367) | 92% (615) |
| Leukocyte esterase > - & Nitrite + | 27% (2,055) | 96% (12,620) | 12% (2,520) | 73% (5,576) | 94% (624) |
| Leukocyte esterase > trace & Nitrite + | 25% (1,929) | 97% (12,655) | 11% (2,359) | 75% (5,702) | 94% (627) |
| Leukocyte esterase > small & Nitrite + | 22% (1,686) | 97% (12,719) | 10% (2,052) | 78% (5,945) | 95% (634) |
| Leukocyte esterase > moderate & Nitrite + | 17% (1,272) | 98% (12,809) | 7% (1,548) | 84% (6,359) | 97% (645) |

Supplemental Table 13. **Inpatient Population** Urinalysis Microscopic Results’ Sensitivity & Specificity Analysis WBC Combined with Bacteria (n=17,488)

| WBC (WBCs/HPF) &  >Bacteria none seen (HPF)  count on urine microscope  **ROC Curve (AUC=0.722)** | Sensitivity for detecting a uropathogen  n=5,976 | Specificity for detecting a uropathogen  n=11,512 | % of ordered  urine cultures that would be  processed  n=17,488 | % of uropathogens that would be  excluded from culture  n=5,976 | % of non-uropathogens that would be  excluded from culture  n=842 |
| --- | --- | --- | --- | --- | --- |
| WBC >0 & >Bacteria none seen | 100% (5,976) | 0% (0) | 100% (17,488) | 0% (0) | 0% (0) |
| WBC >3 & >Bacteria none seen | 99% (5925) | 2% (228) | 98% (17,209) | 1% (51) | 0.5% (4) |
| WBC >10 & >Bacteria none seen | 83% (4,969) | 45% (5,209) | 65% (11,272) | 17% (1,007) | 22% (178) |
| WBC >30 & >Bacteria none seen | 69% (4,080) | 68% (7,825) | 45% (7,767) | 32% (1,896) | 42% (352) |
| WBC >50 & >Bacteria none seen | 52% (3,095) | 82% (9,422) | 30% (5,185) | 48% (2,881) | 61% (510) |
| WBC >50 & >Bacteria none seen | 42% (2,530) | 87% (10,007) | 23% (4,035) | 58% (3,446) | 70% (591) |

| WBC (WBCs/HPF) &  > few bacteria (HPF)  count on urine microscope  **ROC Curve (AUC=0.714)** | Sensitivity for detecting a uropathogen  n=5,976 | Specificity for detecting a uropathogen  n=11,512 | % of ordered  urine cultures that would be  processed  n=17,488 | % of uropathogens that would be  excluded from culture  n=5,976 | % of non-uropathogens that would be  excluded from culture  n=842 |
| --- | --- | --- | --- | --- | --- |
| WBC >0 & >Bacteria few | 84% (5,001) | 37% (4,354) | 70% (12,159) | 16% (975) | 26% (219) |
| WBC >3 & > Bacteria few | 83% (4,986) | 38% (4,406) | 70% (12,092) | 16% (990) | 26% (220) |
| WBC >10 & > Bacteria few | 75% (4,443) | 57% (6,581) | 54% (9,374) | 25% (1,533) | 37% (310) |
| WBC >30 & > Bacteria few | 63% (3,759) | 73% (8,432) | 39% (6,839) | 37% (2,217) | 52% (433) |
| WBC >50 & > Bacteria few | 49% (2,913) | 84% (9,707) | 27% (4,718) | 51% (3,063) | 68% (569) |
| WBC >50 & > Bacteria few | 40% (2,390) | 89% (10,194) | 21% (3,708) | 60% (3,586) | 74% (636) |

| WBC (WBCs/HPF) &  >Bacteria moderate (HPF)  count on urine microscope  **ROC Curve (AUC=0.662)** | Sensitivity for detecting a uropathogen  n=5,976 | Specificity for detecting a uropathogen  n=11,512 | % of ordered  urine cultures that would be  processed  n=17,488 | % of uropathogens that would be  excluded from culture  n=5,976 | % of non-uropathogens that would be  excluded from culture  n=842 |
| --- | --- | --- | --- | --- | --- |
| WBC >0 & > Bacteria moderate | 46% (2,718) | 84% (9,759) | 26% (4,471) | 54% (3,258) | 80% (678) |
| WBC >3 & >Bacteria moderate | 46% (2,713) | 84% (9,764) | 26% (4,461) | 54% (3,263) | 80% (678) |
| WBC >10 & >Bacteria moderate | 44% (2,588) | 87% (10,002) | 24% (4,098) | 56% (3,388) | 82% (692) |
| WBC >30 & >Bacteria moderate | 39% (2,318) | 90% (10,367) | 20% (3,463) | 61% (3,658) | 86% (721) |
| WBC >50 & >Bacteria moderate | 32% (1,898) | 93% (10,718) | 15% (2,692) | 68% (4,078) | 89% (748) |
| WBC >50 & >Bacteria moderate | 27% (1,609) | 95% (10,900) | 13% (2,221) | 73% (4,367) | 91% (765) |

| WBC (WBCs/HPF) &  Bacteria (HPF)  count on urine microscope  **ROC Curve (AUC=0.614)** | Sensitivity for detecting a uropathogen  n=5,976 | Specificity for detecting a uropathogen  n=11,512 | % of ordered  urine cultures that would be  processed  n=17,488 | % of uropathogens that would be  excluded from culture  n=5,976 | % of non-uropathogens that would be  excluded from culture  n=842 |
| --- | --- | --- | --- | --- | --- |
| WBC >0 & > Bacteria many | 29% (1,695) | 94% (10,830) | 14% (2,378) | 71% (4,280) | 92% (776) |
| WBC >3 & > Bacteria many | 29% (1,694) | 94% (10,835) | 14% (2,371) | 71% (4,282) | 92% (776) |
| WBC >10 & > Bacteria many | 27% (1,621) | 94% (10,878) | 13% (2,255) | 73% (4,355) | 93% (781) |
| WBC >30 & > Bacteria many | 25% (1,466) | 95% (10,984) | 11% (1,994) | 75% (4,510) | 94% (788) |
| WBC >50 & > Bacteria many | 20% (1,204) | 97% (11,128) | 9% (1,588) | 80% (4,772) | 95% (802) |
| WBC >50 & > Bacteria many | 18% (1,043) | 97% (11,196) | 8% (1,359) | 82% (4,933) | 96% (808) |

Supplemental Table 14. **Inpatient Population** Urinalysis Microscopic Results’ Sensitivity & Specificity Analysis WBC Combined with Squamous Epithelial Cells (n=17,488)

| WBC (WBCs/HPF) &  Squamous epithelial cells $\geq0$(LPF)  count on urine microscope  **ROC Curve (AUC=0.722)** | Sensitivity for detecting a uropathogen  n=5,976 | Specificity for detecting a uropathogen  n=11,512 | % of ordered  urine cultures that would be  processed  n=17,488 | % of uropathogens that would be  excluded from culture  n=5,976 | % of non-uropathogens that would be  excluded from culture  n=842 |
| --- | --- | --- | --- | --- | --- |
| WBC >0 & Squamous epithelial cells $\geq0$ | 100% (5,976) | 0% (0) | 100% (17,488) | 0% (0) | 0% (0) |
| WBC >3 & Squamous epithelial cells$\geq0$ | 99% (5,9925) | 2% (228) | 98% (17,209) | 1% (51) | 0.5% (4) |
| WBC >10 & Squamous epithelial cells $\geq0$ | 83% (4,969) | 45% (5,209) | 65% (11,272) | 17% (1,007) | 21% (178) |
| WBC >30 & Squamous epithelial cells $\geq0$ | 68% (4,080) | 68% (7,825) | 44% (7,767) | 32% (1,896) | 42% (352) |
| WBC >50 & Squamous epithelial cells$\geq0$ | 52% (3,095) | 82% (9,422) | 30% (5,185) | 48% (2,881) | 61% (510) |
| WBC >50 & Squamous epithelial cells $\geq0$ | 42% (2,530) | 87% (10,007) | 23% (4,035) | 58% (3,446) | 70% (591) |

| WBC (WBCs/HPF) &  Squamous epithelial cells $\leq30$ (LPF)  count on urine microscope  **ROC Curve (AUC=0.712)** | Sensitivity for detecting a uropathogen  n=5,976 | Specificity for detecting a uropathogen  n=11,512 | % of ordered  urine cultures that would be  processed  n=17,488 | % of uropathogens that would be  excluded from culture  n=5,976 | % of non-uropathogens that would be  excluded from culture  n=842 |
| --- | --- | --- | --- | --- | --- |
| WBC >0 & Squamous epithelial cells $\leq30$ | 76% (4,517) | 22% (2,547) | 77% (13,482) | 24% (1,459) | 25% (214) |
| WBC >3 & Squamous epithelial cells $\leq30$ | 75% (4,473) | 24% (2,748) | 76% (13,237) | 25% (1,503) | 26% (217) |
| WBC >10 & Squamous epithelial cells $\leq30$ | 62% (3,687) | 62% (7,110) | 46% (8,089) | 38% (2,289) | 42% (358) |
| WBC >30 & Squamous epithelial cells $\leq30$ | 51% (3,030) | 79% (9,060) | 31% (5,482) | 49% (2,946) | 57% (476) |
| WBC >50 & Squamous epithelial cells $\leq30$ | 38% (2,296) | 88% (10,145) | 21% (3,663) | 62% (3,680) | 70% (593) |
| WBC >50 & Squamous epithelial cells $\leq30$ | 32% (1,901) | 91% (10,516) | 17% (2,897) | 68% (4,075) | 77% (648) |

| WBC (WBCs/HPF) &  Squamous epithelial cells $\leq15$ (LPF)  count on urine microscope  **ROC Curve (AUC=0.700)** | Sensitivity for detecting a uropathogen  n=5,976 | Specificity for detecting a uropathogen  n=11,512 | % of ordered  urine cultures that would be  processed  n=17,488 | % of uropathogens that would be  excluded from culture  n=5,976 | % of non-uropathogens that would be  excluded from culture  n=842 |
| --- | --- | --- | --- | --- | --- |
| WBC >0 & Squamous epithelial cells $\leq15$ | 63% (3,759) | 33% (3,845) | 65% (11,426) | 37% (2,217) | 37% (309) |
| WBC >3 & Squamous epithelial cells $\leq15$ | 62% (3,716) | 35% (4,037) | 64% (11,191) | 38% (2,260) | 37% (312) |
| WBC >10 & Squamous epithelial cells $\leq15$ | 51% (3,042) | 69% (7,990) | 38% (6,564) | 49% (2,934) | 52% (435) |
| WBC >30 & Squamous epithelial cells $\leq15$ | 42% (2,516) | 83% (9,574) | 26% (4,454) | 58% (3,460) | 64% (539) |
| WBC >50 & Squamous epithelial cells $\leq15$ | 32% (1,922) | 91% (10,432) | 17% (3,002) | 68% (4,054) | 75% (631) |
| WBC >50 & Squamous epithelial cells $\leq15$ | 27% (1,604) | 93% (10,712) | 14% (2,404) | 73% (4,372) | 80% (676) |

| WBC (WBCs/HPF) &  & Squamous epithelial cells $\leq3$ (LPF)  count on urine microscope  **ROC Curve (AUC=0.651)** | Sensitivity for detecting a uropathogen  n=5,976 | Specificity for detecting a uropathogen  n=11,512 | % of ordered  urine cultures that would be  processed  n=17,488 | % of uropathogens that would be  excluded from culture  n=5,976 | % of non-uropathogens that would be  excluded from culture  n=842 |
| --- | --- | --- | --- | --- | --- |
| WBC >0 & Squamous epithelial cells $\leq3$ | 37% (2,227) | 57% (6,549) | 41% (7,190) | 63% (3,749) | 60% (500) |
| WBC >3 & Squamous epithelial cells $\leq3$ | 37% (2,194) | 58% (6,703) | 40% (7,003) | 63% (3,782) | 60% (503) |
| WBC >10 & Squamous epithelial cells $\leq3$ | 30% (1,786) | 82% (9,438) | 22% (3,860) | 70% (4,190) | 67% (563) |
| WBC >30 & Squamous epithelial cells $\leq3$ | 25% (1,517) | 90% (10,328) | 15% (2,701) | 75% (4,459) | 75% (627) |
| WBC >50 & Squamous epithelial cells $\leq3$ | 19% (1,163) | 94% (10,843) | 11% (1,832) | 81% (4,813) | 83% (696) |
| WBC >50 & Squamous epithelial cells $\leq3$ | 17% (988) | 96% (11,007) | 9% (1,493) | 83% (4,988) | 86% (723) |

| WBC (WBCs/HPF) &  & Squamous epithelial cells $=0$(LPF)  count on urine microscope  **ROC Curve (AUC=0.607)** | Sensitivity for detecting a uropathogen  n=5,976 | Specificity for detecting a uropathogen  n=11,512 | % of ordered  urine cultures that would be  processed  n=17,488 | % of uropathogens that would be  excluded from culture  n=5,976 | % of non-uropathogens that would be  excluded from culture  n=842 |
| --- | --- | --- | --- | --- | --- |
| WBC >0 & Squamous epithelial cells $=0$ | 23% (1,380) | 74% (8,454) | 25% (4,438) | 77% (4,596) | 73% (612) |
| WBC >3 & Squamous epithelial cells $=0$ | 23% (1,362) | 74% (8,559) | 25% (4,315) | 77% (4,614) | 73% (613) |
| WBC >10 & Squamous epithelial cells $=0$ | 19% (1,139) | 89% (10,241) | 14% (2,410) | 81% (4,837) | 77% (650) |
| WBC >30 & Squamous epithelial cells $=0$ | 17% (999) | 94% (10,764) | 10% (1,747) | 83% (4,977) | 82% (686) |
| WBC >50 & Squamous epithelial cells $=0$ | 13% (785) | 96% (11,066) | 7% (1,231) | 87% (5,191) | 87% (728) |
| WBC >50 & Squamous epithelial cells $=0$ | 11% (679) | 97% (11,178) | 6% (1,013) | 89% (5,297) | 89% (747) |

Supplemental Table 15. **Inpatient Population** Urinalysis Microscopic Results’ Sensitivity & Specificity Analysis Leukocyte Esterase Combined with Nitrite (n=17,488)

| Urinalysis Leukocyte esterase &  Urinalysis Nitrite - or +  value on urine macroscopic examination  **ROC Curve (AUC=0.719)** | Sensitivity for detecting a uropathogen  n=5,976 | Specificity for detecting a uropathogen  n=11,512 | % of ordered  urine cultures that would be  processed  n=17,488 | % of uropathogens that would be  excluded from culture  n=5,976 | % of non-uropathogens that would be  excluded from culture  n=842 |
| --- | --- | --- | --- | --- | --- |
| Leukocyte esterase > - & Nitrite - or + | 100% (5,976) | 0% (0) | 100% (17,488) | 0% (0) | 0% (0) |
| Leukocyte esterase > trace & Nitrite - or + | 80% (4,764) | 53% (6,066) | 58% (10,210) | 20% (1,212) | 27% (228) |
| Leukocyte esterase > small & Nitrite - or + | 73% (4,324) | 63% (7,194) | 50% (8,642) | 27% (1,652) | 36% (299) |
| Leukocyte esterase > moderate & Nitrite - or + | 61% (3,653) | 74% (8,557) | 38% (6,608) | 39% (2,323) | 52% (435) |
| Leukocyte esterase > moderate & Nitrite - or + | 46% (2,725) | 85% (9,793) | 25% (4,444) | 54% (3,251) | 67% (566) |

| Urinalysis Leukocyte esterase &  Urinalysis Nitrite +  value on urine macroscopic examination  **ROC Curve (AUC=0.620)** | Sensitivity for detecting a uropathogen  n=5,976 | Specificity for detecting a uropathogen  n=11,512 | % of ordered  urine cultures that would be  processed  n=17,488 | % of uropathogens that would be  excluded from culture  n=5,976 | % of non-uropathogens that would be  excluded from culture  n=842 |
| --- | --- | --- | --- | --- | --- |
| Leukocyte esterase > - & Nitrite + | 29% (1,742) | 94% (10,875) | 14% (2,379) | 71% (4,234) | 94% (789) |
| Leukocyte esterase > - & Nitrite + | 27% (1,598) | 95% (10,955) | 12% (2,155) | 73% (4,378) | 95% (797) |
| Leukocyte esterase > trace & Nitrite + | 25% (1,513) | 96% (10,997) | 12% (2,028) | 75% (4,463) | 95% (801) |
| Leukocyte esterase > small & Nitrite + | 22% (1,339) | 96% (11,105) | 10% (1,746) | 78% (4,637) | 96% (810) |
| Leukocyte esterase > moderate & Nitrite + | 18% (1,053) | 98% (11,229) | 8% (1,336) | 82% (4,923) | 97% (818) |

Supplemental Table 16. **Outpatient Population** Urinalysis Microscopic Results’ Sensitivity & Specificity Analysis WBC Combined with Bacteria (n=42,745)

| WBC (WBCs/HPF) &  >Bacteria none seen (HPF)  count on urine microscope  **ROC Curve (AUC=0.720)** | Sensitivity for detecting a uropathogen  n=15,386 | Specificity for detecting a uropathogen  n=27,359 | % of ordered  urine cultures that would be  processed  n=42,745 | % of uropathogens that would be  excluded from culture  n=15,386 | % of non-uropathogens that would be  excluded from culture  n=1,450 |
| --- | --- | --- | --- | --- | --- |
| WBC >0 & >Bacteria none seen | 100% (15,484) | 0% (0) | 100% (42,745) | 0% (0) | 0% (0) |
| WBC >3 & >Bacteria none seen | 99% (15,251) | 3% (674) | 98% (41,936) | 1% (135) | 1% (15) |
| WBC >10 & >Bacteria none seen | 80% (12,291) | 50% (13,683) | 61% (25,967) | 20% (3,095) | 37% (537) |
| WBC >30 & >Bacteria none seen | 61% (9,386) | 75% (20,428) | 38% (16,317) | 39% (6,000) | 65% (938) |
| WBC >50 & >Bacteria none seen | 43% (6,589) | 88% (23,943) | 23% (10,005) | 57% (8,797) | 80% (1,163) |
| WBC >50 & >Bacteria none seen | 35% (5,366) | 91% (24,984) | 18% (7,741) | 65% (10,020) | 85% (1,239) |

| WBC (WBCs/HPF) &  > few bacteria (HPF)  count on urine microscope  **ROC Curve (AUC=0.709)** | Sensitivity for detecting a uropathogen  n=15,386 | Specificity for detecting a uropathogen  n=27,359 | % of ordered  urine cultures that would be  processed  n=42,745 | % of uropathogens that would be  excluded from culture  n=15,386 | % of non-uropathogens that would be  excluded from culture  n=1,450 |
| --- | --- | --- | --- | --- | --- |
| WBC >0 & >Bacteria few | 78% (12,070) | 43% (11,790) | 65% (27,639) | 22% (3,316) | 28% (410) |
| WBC >3 & > Bacteria few | 78% (12,038) | 44% (11,935) | 64% (27,462) | 22% (3,348) | 29% (416) |
| WBC >10 & > Bacteria few | 69% (10,675) | 63% (17,212) | 49% (20,822) | 31% (4,711) | 49% (707) |
| WBC >30 & > Bacteria few | 55% (8,476) | 80% (21,829) | 33% (14,006) | 45% (6,910) | 70% (1,016) |
| WBC >50 & > Bacteria few | 40% (6094) | 89% (24,465) | 21% (8,988) | 60% (9,292) | 83% (1,201) |
| WBC >50 & > Bacteria few | 32% (4,990) | 93% (25,304) | 17% (7,045) | 68% (10,396) | 87% (1,266) |

| WBC (WBCs/HPF) &  >Bacteria moderate (HPF)  count on urine microscope  **ROC Curve (AUC=0.622)** | Sensitivity for detecting a uropathogen  n=15,386 | Specificity for detecting a uropathogen  n=27,359 | % of ordered  urine cultures that would be  processed  n=42,745 | % of uropathogens that would be  excluded from culture  n=15,386 | % of non-uropathogens that would be  excluded from culture  n=1,450 |
| --- | --- | --- | --- | --- | --- |
| WBC >0 & > Bacteria moderate | 34% (5,179) | 90% (24,513) | 19% (8,025) | 66% (10,207) | 82% (1,192) |
| WBC >3 & >Bacteria moderate | 34% (5,168) | 90% (24,527) | 19% (8,000) | 66% (10,218) | 82% (1,193) |
| WBC >10 & >Bacteria moderate | 32% (4,841) | 92% (25,055) | 17% (7,145) | 69% (10,545) | 85% (1,245) |
| WBC >30 & >Bacteria moderate | 27% (4,098) | 94% (25,830) | 13% (5,627) | 73% (11,288) | 92% (1,335) |
| WBC >50 & >Bacteria moderate | 20% (3,129) | 96% (26,374) | 10% (4,114) | 80% (12,257) | 95% (1,381) |
| WBC >50 & >Bacteria moderate | 17% (2,632) | 97% (26,613) | 8% (3,378) | 83% (12,754) | 86% (1,398) |

| WBC (WBCs/HPF) &  Bacteria (HPF)  count on urine microscope  **ROC Curve (AUC=0.575)** | Sensitivity for detecting a uropathogen  n=15,386 | Specificity for detecting a uropathogen  n=27,359 | % of ordered  urine cultures that would be  processed  n=42,745 | % of uropathogens that would be  excluded from culture  n=15,386 | % of non-uropathogens that would be  excluded from culture  n=1,450 |
| --- | --- | --- | --- | --- | --- |
| WBC >0 & > Bacteria many | 18% (2,836) | 96% (26,376) | 9% (3,819) | 82% (12,550) | 95% (1,378) |
| WBC >3 & > Bacteria many | 18% (2,830) | 96% (26,380) | 9% (3,809) | 82% (12,556) | 95% (1,378) |
| WBC >10 & > Bacteria many | 17% (2,670) | 97% (26,501) | 8% (3,528) | 83% (12,716) | 96% (1,389) |
| WBC >30 & > Bacteria many | 15% (2,247) | 98% (26,725) | 7% (2,881) | 85% (13,139) | 97% (1,413) |
| WBC >50 & > Bacteria many | 11% (1,755) | 98% (26,918) | 5% (2,196) | 89% (13,631) | 98% (1,429) |
| WBC >50 & > Bacteria many | 10% (1,511) | 99% (27,147) | 4% (1,864) | 90% (13,973) | 99% (1,433) |

Supplemental Table 17. **Outpatient Population** Urinalysis Microscopic Results’ Sensitivity & Specificity Analysis WBC Combined with Squamous Epithelial Cells (n=42,745)

| WBC (WBCs/HPF) &  Squamous epithelial cells $\geq0$(LPF)  count on urine microscope  **ROC Curve (AUC=0.720)** | Sensitivity for detecting a uropathogen  n=15,386 | Specificity for detecting a uropathogen  n=27,359 | % of ordered  urine cultures that would be  processed  n=42,745 | % of uropathogens would be  excluded from culture  n=15,386 | % of non-uropathogens would be  excluded from culture  n=1,450 |
| --- | --- | --- | --- | --- | --- |
| WBC >0 & Squamous epithelial cells $\geq0$ | 100% (15,386) | 0% (0) | 100% (42,745) | 0% (0) | 0% (0) |
| WBC >3 & Squamous epithelial cells$\geq0$ | 99% (15,251) | 2% (674) | 98% (41,936) | 1% (135) | 1% (15) |
| WBC >10 & Squamous epithelial cells $\geq0$ | 80% (12,291) | 50% (13,683) | 61% (25,967) | 20% (3,095) | 37% (537) |
| WBC >30 & Squamous epithelial cells $\geq0$ | 61% (9,386) | 75% (20,428) | 38% (16,317) | 39% (6,000) | 65% (938) |
| WBC >50 & Squamous epithelial cells$\geq0$ | 43% (6,589) | 88% (23,943) | 23% (10,005) | 57% (8,797) | 80% (1,163) |
| WBC >50 & Squamous epithelial cells $\geq0$ | 35% (5,366) | 91% (4,984) | 18% (7,741) | 65% (10,020) | 85% (1,239) |

| WBC (WBCs/HPF) &  Squamous epithelial cells $\leq30$ (LPF)  count on urine microscope  **ROC Curve (AUC=0.680)** | Sensitivity for detecting a uropathogen  n=15,386 | Specificity for detecting a uropathogen  n=27,359 | % of ordered  urine cultures that would be  processed  n=42,745 | % of uropathogens would be  excluded from culture  n=15,386 | % of non-uropathogens would be  excluded from culture  n=1,450 |
| --- | --- | --- | --- | --- | --- |
| WBC >0 & Squamous epithelial cells $\leq30$ | 55% (15,153) | 39% (6,058) | 57% (24,481) | 45% (12,206) | 58% (840) |
| WBC >3 & Squamous epithelial cells $\leq30$ | 53% (14,588) | 40% (6,157) | 56% (23,817) | 47% (12,771) | 59% (851) |
| WBC >10 & Squamous epithelial cells $\leq30$ | 22% (6,030) | 53% (8,095) | 31% (13,321) | 78% (21,329) | 76% (1,105) |
| WBC >30 & Squamous epithelial cells $\leq30$ | 12% (3,236) | 63% (9,697) | 21% (8,925) | 88% (24,123) | 83% (1,208) |
| WBC >50 & Squamous epithelial cells $\leq30$ | 7% (1,798) | 73% (11,290) | 14% (5,894) | 93% (25,561) | 89% (1,285) |
| WBC >50 & Squamous epithelial cells $\leq30$ | 5% (1,327) | 78% (12,012) | 11% (4,701) | 95% (26,032) | 91% (1,320) |

| WBC (WBCs/HPF) &  Squamous epithelial cells $\leq15$ (LPF)  count on urine microscope  **ROC Curve (AUC=0.646)** | Sensitivity for detecting a uropathogen  n=15,386 | Specificity for detecting a uropathogen  n=27,359 | % of ordered  urine cultures that would be  processed  n=42,745 | % of uropathogens would be  excluded from culture  n=15,386 | % of non-uropathogens would be  excluded from culture  n=1,450 |
| --- | --- | --- | --- | --- | --- |
| WBC >0 & Squamous epithelial cells $\leq15$ | 44% (6,798) | 58% (15,858) | 43% (18,299) | 55% (8,588) | 70% (1,019) |
| WBC >3 & Squamous epithelial cells $\leq15$ | 44% (6,719) | 60% (16,358) | 41% (17,720) | 56% (8,667) | 71% (1,028) |
| WBC >10 & Squamous epithelial cells $\leq15$ | 34% (5,264) | 84% (23,053) | 22% (9,570) | 66% (10,122) | 82% (1,194) |
| WBC >30 & Squamous epithelial cells $\leq15$ | 27% (4,161) | 91% (24,962) | 15% (6,558) | 73% (11,225) | 86% (1,253) |
| WBC >50 & Squamous epithelial cells $\leq15$ | 20% (3,027) | 95% (25,988) | 10% (4,398) | 80% (12,359) | 90% (1,312) |
| WBC >50 & Squamous epithelial cells $\leq15$ | 16% (2,527) | 96% (26,331) | 8% (3,555) | 83% (12,859) | 92% (1,338) |

| WBC (WBCs/HPF) &  & Squamous epithelial cells $\leq3$ (LPF)  count on urine microscope  **ROC Curve (AUC=0.588)** | Sensitivity for detecting a uropathogen  n=15,386 | Specificity for detecting a uropathogen  n=27,359 | % of ordered  urine cultures that would be  processed  n=42,745 | % of uropathogens would be  excluded from culture  n=15,386 | % of non-uropathogens would be  excluded from culture  n=1,450 |
| --- | --- | --- | --- | --- | --- |
| WBC >0 & Squamous epithelial cells $\leq3$ | 22% (3,300) | 77% (20,951) | 23% (9,708) | 79% (12,086) | 85% (1,235) |
| WBC >3 & Squamous epithelial cells $\leq3$ | 21% (3,241) | 78% (21,307) | 22% (9,293) | 79% (12,145) | 85% (1,238) |
| WBC >10 & Squamous epithelial cells $\leq3$ | 16% (2,527) | 92% (25,195) | 11% (4,691) | 84% (12,859) | 90% (1,310) |
| WBC >30 & Squamous epithelial cells $\leq3$ | 13% (2,043) | 95% (26,102) | 8% (3,300) | 87% (13,343) | 93% (1,342) |
| WBC >50 & Squamous epithelial cells $\leq3$ | 10% (1,514) | 97% (26,653) | 5% (2,245) | 90% (13,909) | 95% (1,375) |
| WBC >50 & Squamous epithelial cells $\leq3$ | 8% (1,258) | 98% (26,826) | 4% (1,816) | 92% (14,165) | 96% (1,390) |

| WBC (WBCs/HPF) &  & Squamous epithelial cells $=0$ (LPF)  count on urine microscope  **ROC Curve (AUC=0.563)** | Sensitivity for detecting a uropathogen  n=15,386 | Specificity for detecting a uropathogen  n=27,359 | % of ordered  urine cultures that would be  processed  n=42,745 | % of uropathogens would be  excluded from culture  n=15,386 | % of non-uropathogens would be  excluded from culture  n=1,450 |
| --- | --- | --- | --- | --- | --- |
| WBC >0 & Squamous epithelial cells $=0$ | 13% (2,055) | 85% (23,213) | 15% (6,201) | 87% (13,331) | 92% (1,327) |
| WBC >3 & Squamous epithelial cells $=0$ | 13% (2,006) | 86% (23,490) | 14% (5,875) | 87% (13,380) | 92% (1,329) |
| WBC >10 & Squamous epithelial cells $=0$ | 11% (1,628) | 95% (25,992) | 7% (2,995) | 89% (13,758) | 94% (1,369) |
| WBC >30 & Squamous epithelial cells $=0$ | 9% (1,366) | 97% (26,517) | 5% (2,202) | 91% (14,026) | 95% (1,383) |
| WBC >50 & Squamous epithelial cells $=0$ | 7% (1,051) | 98% (26,853) | 4% (1,557) | 93% (14,335) | 97% (1,402) |
| WBC >50 & Squamous epithelial cells $=0$ | 6% (899) | 99% (26,959) | 3% (1,299) | 94% (14,487) | 97% (1,408) |

Supplemental Table 18. **Outpatient Population** Urinalysis Microscopic Results’ Sensitivity & Specificity Analysis: Leukocyte Esterase Combined with Nitrite (n=42,745)

| Urinalysis Leukocyte esterase &  Urinalysis Nitrite - or +  value on urine macroscopic examination  **ROC Curve (AUC=0.681)** | Sensitivity for detecting a uropathogen  n=15,386 | Specificity for detecting a uropathogen  n=27,359 | % of ordered  urine cultures that would be  processed  n=42,745 | % of uropathogens that would be  excluded from culture  n=15,386 | % of non-uropathogens that would be  excluded from culture  n=1,450 |
| --- | --- | --- | --- | --- | --- |
| Leukocyte esterase > - & Nitrite - or + | 100% (15,386) | 0% (0) | 100% (42,745) | 0% (0) | 0% (0) |
| Leukocyte esterase > trace & Nitrite - or + | 82% (12,603) | 43% (11,727) | 66% (28,235 | 18% (2,783) | 30% (441) |
| Leukocyte esterase > small & Nitrite - or + | 72% (11,042) | 56% (15,171) | 54% (23,230) | 28% (4,344) | 42% (614) |
| Leukocyte esterase > moderate & Nitrite - or + | 58% (8,886) | 70% (19,226) | 40% (17,019) | 42% (6,500) | 58% (838) |
| Leukocyte esterase > moderate & Nitrite - or + | 40% (6,163) | 84% (22,863) | 25% (10,659) | 60% (9,23) | 74% (1,069) |

| Urinalysis Leukocyte esterase &  Urinalysis Nitrite +  value on urine macroscopic examination  **ROC Curve (AUC=0.632)** | Sensitivity for detecting a uropathogen  n=15,386 | Specificity for detecting a uropathogen  n=27,359 | % of ordered  urine cultures that would be  processed  n=42,745 | % of uropathogens that would be  excluded from culture  n=15,386 | % of non-uropathogens that would be  excluded from culture  n=1,450 |
| --- | --- | --- | --- | --- | --- |
| Leukocyte esterase > - & Nitrite + | 29% (4,516) | 97% (26,530) | 13% (5,345) | 71% (10,870) | 95% (1,371) |
| Leukocyte esterase > - & Nitrite + | 26% (4,072) | 98% (26,700) | 11% (4,731) | 74% (11,314) | 95% (1,382) |
| Leukocyte esterase > trace & Nitrite + | 24% (3,759) | 98% (26,735) | 10% (4,383) | 76% (11,627) | 96% (1,388) |
| Leukocyte esterase > small & Nitrite + | 21% (3,181) | 98% (26,818) | 9% (3,722) | 79% (12,205) | 97% (1,401) |
| Leukocyte esterase > moderate & Nitrite + | 16% (2,405) | 99% (26,956) | 7% (2,799) | 84% (2,981) | 98% (1,417) |
